# AAV9-Tspyl2 gene therapy retards bleomycin-induced pulmonary fibrosis by modulating downstream TGF-β signaling in mice

**DOI:** 10.1038/s41419-023-05889-8

**Published:** 2023-06-30

**Authors:** Shijie Zhang, Xiang Tong, Sitong Liu, Jizhen Huang, Li Zhang, Tianli Zhang, Dongguang Wang, Hong Fan

**Affiliations:** Department of Respiratory and Critical Care Medicine, West China Hospital/West China School of Medicine, Sichuan University, Chengdu, China

**Keywords:** Chronic inflammation, Respiratory tract diseases

## Abstract

Idiopathic pulmonary fibrosis (IPF) is a devastating fibrotic lung disease characterized by scarring and destruction of the lung architecture, with limited treatment options. Targeted gene therapy to restore cell division autoantigen-1 (CDA1) expression may be a potential treatment approach to delay the progression of pulmonary fibrosis (PF). Here, we focused on CDA1, which was significantly decreased in human IPF, in a mouse model of bleomycin (BLM)-induced PF, and in transforming growth factor (TGF-β)-challenged lung fibroblasts. In vitro, CDA1 overexpression by lentivirus infection in human embryonic lung fibroblasts (HFL1 cells) inhibited the production of pro-fibrotic and pro-inflammatory cytokines, lung fibroblast-to-myofibroblast transition, and extracellular matrix protein expression induced by exogenous TGF‐β1 treatment, whereas CDA1 knockdown with small interfering RNA promoted this effect. CDA1 overexpression also inhibited cell proliferation and migration. In a mouse model of BLM-induced PF, we provided novel evidence that the intratracheal delivery of adeno-associated virus serotype 9 carrying the mouse *Tspyl2* gene reduced lung tissue inflammation and fibrosis. Mechanistically, CDA1, as a transcription regulator, could repress the TGF-β signal transduction in vivo and in vitro. In conclusion, our results show that *Tspyl2* gene therapy plays an antifibrotic role by inhibiting the lung fibroblast-to-myofibroblast transition and downstream TGF-β/Smad3 signaling transduction in BLM-induced PF in mice, suggesting that CDA1 is an appropriate and promising therapeutic target for PF.

## Introduction

Idiopathic pulmonary fibrosis (IPF) is a chronic progressive interstitial lung disease with unknown causes, characterized by scarring and destruction of the lung architecture, leading to reduced quality of life and early death [1]. From 2009–2020, the incidence of IPF (per 10,000 of the population) ranged from 0.35-1.30 in Asia-Pacific countries, 0.09-0.49 in Europe, and 0.75-0.93 in North America [2]. Although IPF remains rare, its incidence has gradually increased [3].The median survival time of IPF from diagnosis is only 2-4 years [4], which is comparable to that of invasive tumors and seriously threatens the life and health of patients. Currently, the only drugs approved internationally for IPF treatment are pirfenidone and nintedanib [5]; however, neither of these drugs can reduce respiratory-specific mortality and all-cause mortality [6]. Thus, there is an urgent need to identify novel targets that are safe and effective for IPF treatment.

The pathogenesis of IPF is complex and multifactorial, and involves the precise regulation of various types of cells, inflammatory factors, chemokines, and pro-fibrotic factors [7]. Among them, lung myofibroblasts are the key effector cells, which are mainly derived from the transdifferentiation of lung fibroblasts and are responsible for the synthesis and deposition of the extracellular matrix (ECM), resulting in the loss of alveolar function and structural remodeling [8]. Transdifferentiation and proliferation of lung myofibroblasts are the key factors in progressive fibrosis. Transformation growth factor-β1 (TGF-β1) has long been considered a key pro-fibrotic mediator in organ fibrosis, largely by activating its downstream small mother against decapentaplegic (Smad) signaling pathway [9]. Nonetheless, direct targeted intervention by TGF-β, which is involved in regulating important processes such as embryogenesis and homeostasis, will lead to serious adverse events and even death [10].

Cell division autoantigen-1 (CDA1, also known as TSPYL2, TSPX, DENTT, Se20-4, NP79, CINAP, and CTCL) is a phosphorylated protein encoded by the *Tspyl2* gene on the X chromosome, mainly located in the nucleus, and is widely expressed in various organs and tissues [11, 12]. Initially, CDA1 was identified as a target gene involved in TGF-β1-mediated responses in human lung cancer cells [13]. Recent studies have reported that CDA1 played an important regulatory role in processes including TGF-β signal transduction [11], DNA damage repair [14, 15], cell proliferation [16], gene transcription and translation [12]. Multiple studies by Chai et el. have shown that CDA1 was involved in regulating fibrosis by modulating downstream TGF-β1 signaling in murine diabetic models of atherosclerosis and renal fibrosis, and targeted intervention with CDA1 inhibited the pathological pro-fibrotic effect of TGF-β1 without significant effects on other important physiological functions [17–19]. These studies suggest that CDA1 plays an important role in organ fibrosis. Drug development based on CDA1 is expected to provide new hope for IPF treatment.

Studies have reported that CDA1 is abundantly expressed in lung tissues [20, 21]; however, the role of CDA1 in IPF is unclear. Given the critical role of CDA1 in mediating the biological functions of TGF-β, this study explored the lung effects of targeted intervention with CDA1, with a particular focus on bleomycin (BLM)-induced pulmonary fibrosis (PF), ECM accumulation, lung fibroblast-to-myofibroblast transition, and TGF-β signal transduction.

## Results

### CDA1 expression decreases in human IPF and mouse model of BLM-induced PF

**Fig. 1:**
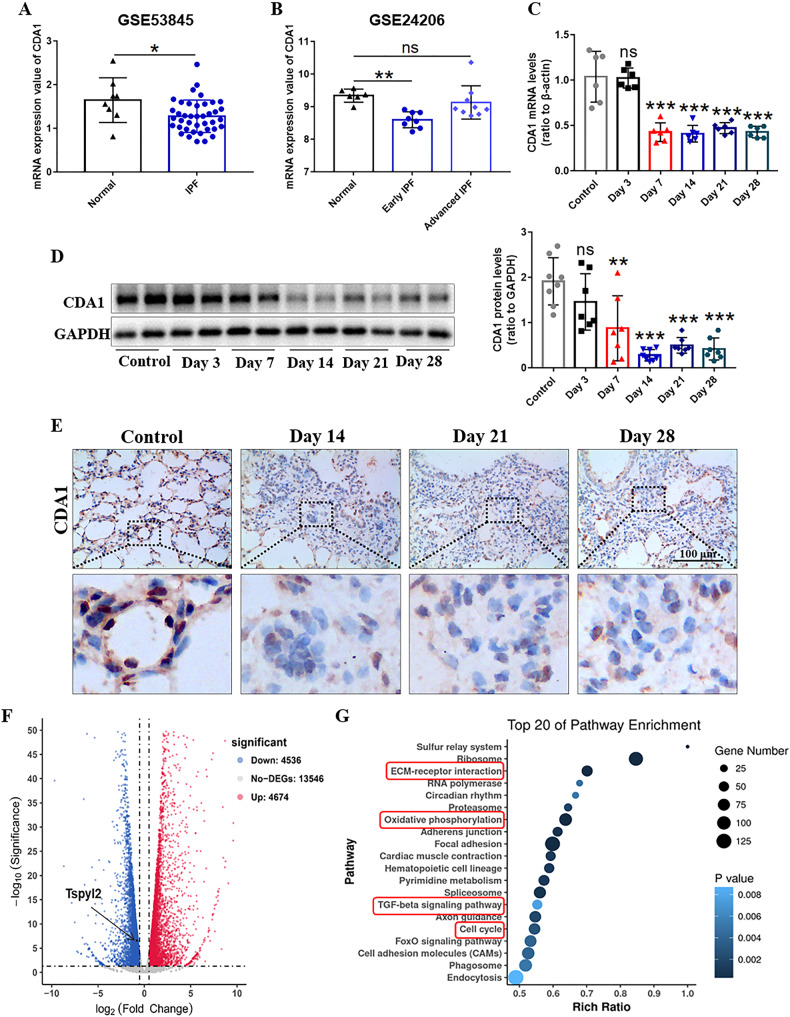
CDA1 expression decreases in human IPF and mouse models of BLM-induced PF. **A**, **B** CDA1 expression level in the lung tissues of IPF and healthy controls in GSE53845 and GSE24206. **C**, **D** The mRNA and protein levels of CDA1 using western blotting and qRT-PCR analysis. **E** Immunohistochemistry staining of CDA1 (dark brown) in mouse lung tissues to assess its expression and cellular localization. **F** Volcano plot of gene expression changes (|log_2_Fold change | >0.5, P < 0.05, FDR < 0.05). **G** KEGG pathway enrichment analysis of all the DEGs. Data are means ± SD; ns no significant difference, *P < 0.05, **P < 0.01, ***P < 0.001. Student’s t test was used in (**A**–**D**). Each group was compared with the control group in (**C**, **D**). DEGs differentially expressed genes. KEGG Kyoto Encyclopedia of Genes and Genomes.

We analyzed the expression of CDA1 in IPF patients in two Gene Expression omnibus (GEO) datasets (GSE53845 and GSE24206). As shown in Fig. 1A, the mRNA expression of CDA1 in the lung tissues of IPF patients was significantly lower than that in healthy controls in GSE53845. Additionally, after differentiating between early and advanced IPF disease stages, the mRNA of CDA1 was significantly decreased in the early stages of IPF patients in GSE24206 (Fig. 1B).

We further evaluated CDA1 level, along with other fibrotic biomarkers, in lung tissues on days 0, 3, 7, 14, 21, and 28 after BLM administration in BLM-induced PF in mice. The mRNA level of α-smooth muscle actin (α-SMA) increased on day 3 and peaked on day 7. The mRNA levels of collagen I, fibronectin, and TGF-β increased on day 7 and peaked on day 14 (Supplementary Fig. 1A). This is consistent with the well-recognized finding that BLM-induced PF exhibits a phase transition from inflammation to fibrosis on day 5-7 [22]. On day 28, the mRNA levels of α-SMA, fibronectin, and TGF-β were still higher than those in the controls, but the collagen I level was not significantly different. In terms of histopathology, Masson and Sirius Red staining revealed obvious morphological changes (thickened alveolar septum and destroyed the alveolar structure) and massive collagen deposition in the lungs on day 14, which were more severe on days 21 and 28 (Supplementary Fig. 1B). Importantly, the mRNA and protein levels of CDA1 decreased significantly on day 7-28 (Fig. 1C–E). Moreover, lung immunohistochemical staining also revealed that CDA1 was mainly expressed in the nucleus (Fig. 1E). Overall, these time-course experiments showed that the expression of CDA1 decreased in parallel with the production of fibrotic biomarkers, and remained low during the fibrotic phase.

To further confirm the changes in CDA1 expression, we performed transcriptome sequencing of fibrotic lung tissues on day 21 in comparison with lung tissues from healthy mice of the same age. A total of 4,536 differentially expressed genes (DEGs) were downregulated in fibrotic lung tissues of mice, including *Tspyl2* (Fig. 1F). Kyoto Encyclopedia of Genes and Genomes (KEGG) pathway analysis of all DEGs revealed the activation of ECM-receptor interaction, TGF-β signaling pathway, cell cycle, and other fibrosis-related pathways (Fig. 1G).

### CDA1 expression decreases after lung fibroblast-to-myofibroblast transition in response to TGF-β1 treatment in vitro

**Fig. 2:**
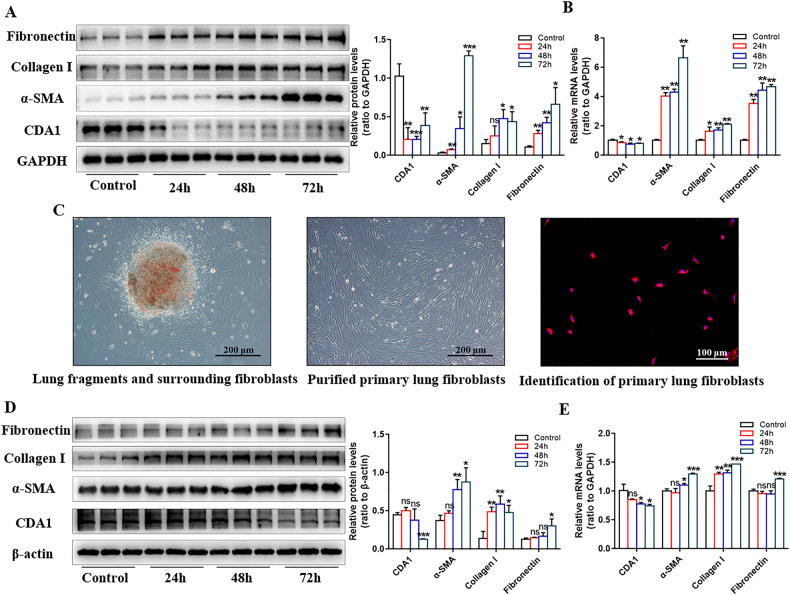
CDA1 expression decreases after the lung fibroblast-to-myofibroblast transition in response to TGF-β treatment in both mouse and human lung fibroblasts. **A**, **B** Time-course analysis of CDA1 and fibrotic biomarkers using western blotting and qRT-PCR analysis in HFL1s in response to TGF-β treatment. **C** Representative images of lung primary fibroblasts purified by crawl out method and identified by vimentin immunofluorescence (red). Nuclei were stained with DAPI (blue). **D**, **E** Time-course analysis of CDA1 and fibrotic biomarkers using western blotting and qRT-PCR analysis in mice lung primary fibroblasts in response to TGF-β treatment. Data are means ± SD; ns no significant difference, *P < 0.05, **P < 0.01, ***P < 0.001. Student’s t test was used in (**A**, **B**, **D**, **E**), and each group was compared with the control group.

TGF-β1 has long been considered a key mediator of organ fibrosis and is an important cytokine involved in the fibroblast-to-myofibroblast transition. Human embryonic lung fibroblasts (HFL1 cells) were stimulated with 10 ng/mL TGF-β1 for 24–72 h. After 24 h of TGF-β1 treatment, the expression of fibrotic biomarkers (α-SMA, collagen I, and fibronectin) began to increase, whereas the mRNA and protein expression of CDA1 decreased (Fig. 2A, B). We also purified and identified murine lung primary fibroblasts (Fig. 2C) and stimulated them with 5 ng/mL TGF-β1 for 24-72 h. After the mouse lung primary fibroblast-to-myofibroblast transition, CDA1 showed a marked decrease, which was significant at 48–72 h (Fig. 2D, E). Overall, these time-course experiments revealed that CDA1 expression decreased in parallel with the production of fibrotic biomarkers in lung fibroblasts in response to TGF-β1 treatment.

### CDA1 overexpression inhibits the expression of fibrotic biomarkers in HFL1 cells

**Fig. 3:**
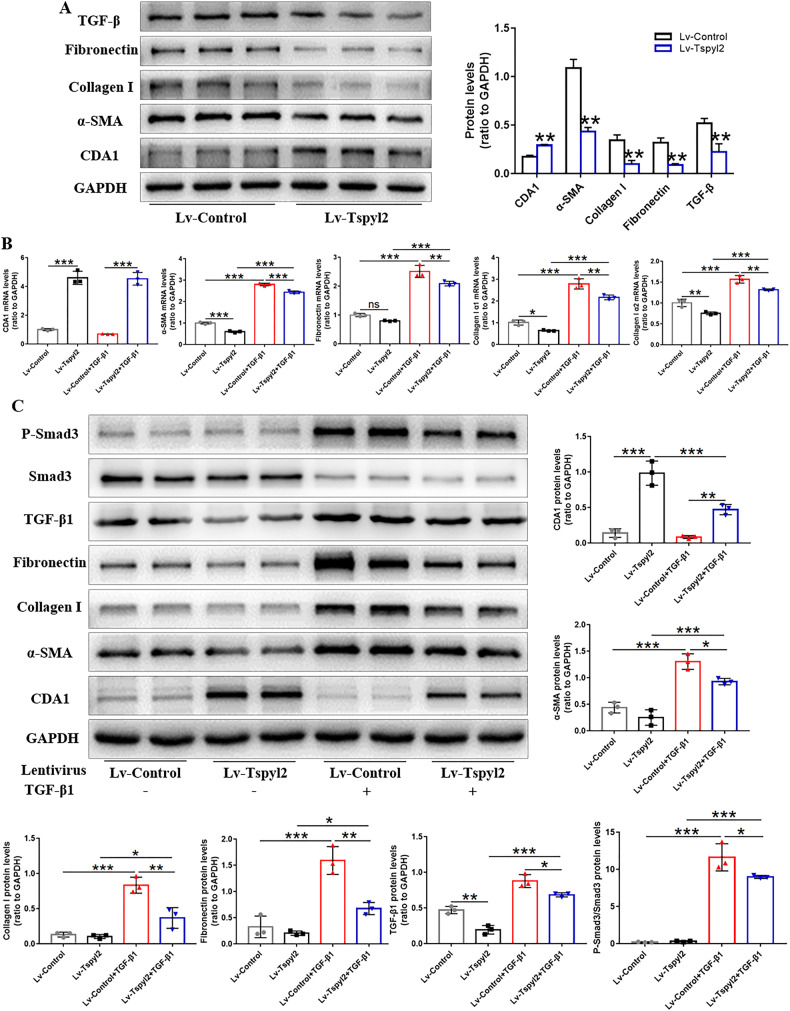
CDA1 overexpression reverses the transdifferentiation of lung fibroblasts into myofibroblasts by abrogating TGF-β/Smad3 signaling in vitro. **A** The protein level of CDA1 and fibrotic biomarkers using western blotting analysis in HFL1 cells after Lv-Tspyl2 infection. **B**, **C** Western blotting and qRT-PCR to test CDA1, fibrotic biomarkers, and TGF-β/Smad3 signaling at protein and mRNA levels after TGF-β1 or Lv-Tspyl2 treatment. Data are means ± SD, ns no significant difference, *P < 0.05, **P < 0.01, ***P < 0.001. Student’s t test was used in (**A**), and one-way ANOVA followed by a Tukey’s test was used in (**B**, **C**).

To confirm the response of fibrotic biomarkers with an increase in CDA1 protein level, the CDA1-encoding gene *Tspyl2* was integrated into the HFL1 cell genome by the lentivirus vector encoding human *Tspyl2* (Lv-Tspyl2) to construct a CDA1-overexpressed HFL1 cell model. Following the increase in CDA1 protein level, the expression of TGF-β, α-SMA, collagen I, and fibronectin was markedly decreased by >50% (Fig. 3A). These findings suggest a physiological role for endogenous CDA1 in the negative regulation of TGF-β and its target genes in HFL1 cells.

### CDA1 overexpression reverses lung fibroblast-to-myofibroblast transition, inflammation, cell proliferation and migration by abrogating TGF-β/Smad3 signaling in vitro

**Fig. 4:**
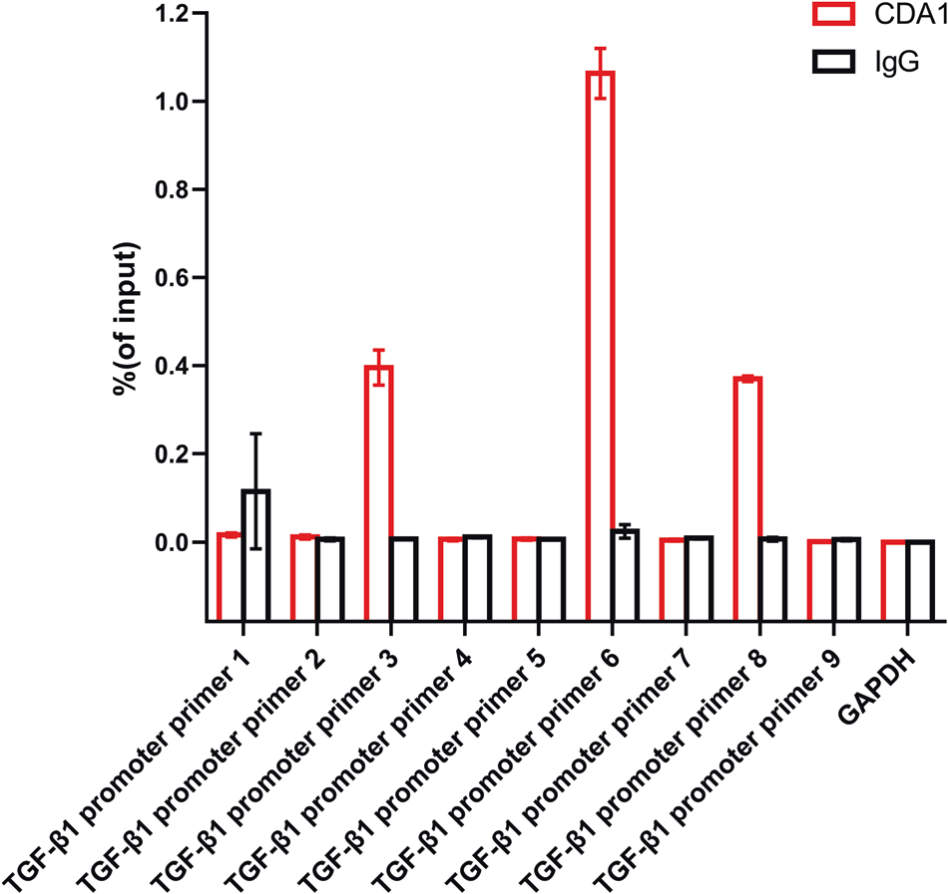
CDA1 is a transcriptional regulator on TGF-β signaling. ChIP analysis was conducted to investigate the transcriptional regulation of CDA1 on TGF-β signaling. Data are means ± SD.

To further confirm the physiological role of CDA1 overexpression, HFL1 cells were infected with Lv-Tspyl2 to upregulate the CDA1 level, after which the cells were stimulated with TGF-β1 (10 ng/mL) for 48 h. The stimulation of TGF-β1 increased α-SMA, collagen I, fibronectin, TGF-β, and P-Smad3/Smad3 expression in HFL1 cells, while CDA1 overexpression blocked this effect (Fig. 3B, C). Moreover, chromatin immunoprecipitation (ChIP) analysis showed a strong occupancy of CDA1 protein with TGF-β1 promoter in the position corresponding to the 6th pair of TGF-β1 promoter primer, and a weak occupancy in the position corresponding to the 3th and 8th pairs of TGF-β1 promoter primers (Fig. 4). These results suggested that CDA1 was a transcriptional regulator on TGF-β signaling.

**Fig. 5:**
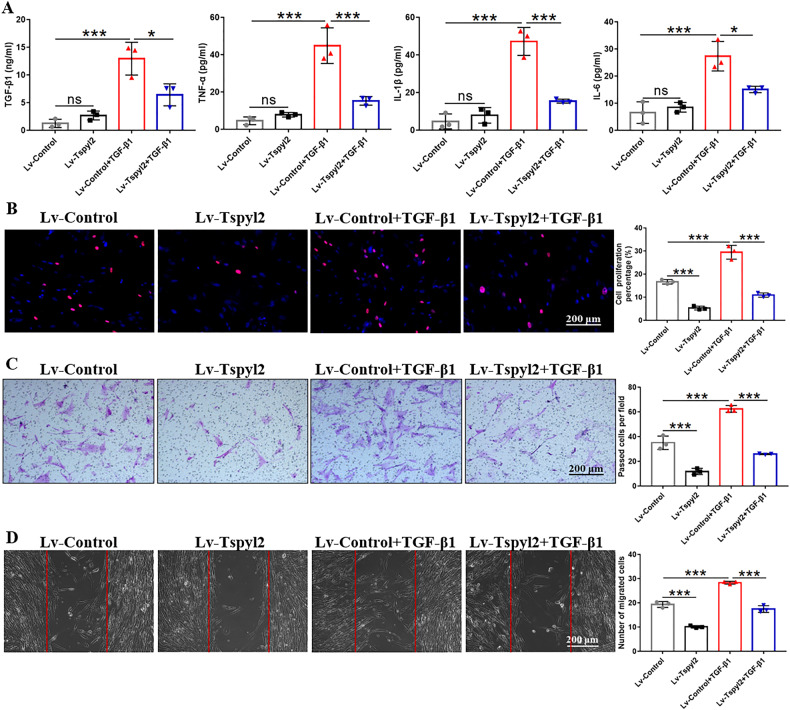
CDA1 overexpression reverses inflammation, cell proliferation, and migration following stimulation with exogenous TGF‐β1 cytokine in HFL1 cells. **A** Measurement of TGF-β1, TNF-α, IL-1β, and IL-6 in the cell culture supernatant by ELISA. **B** EDU staining (red) to detect the influence of CDA1 overexpression on the cell proliferation after TGF‐β1 stimulation. Nuclei were stained with DAPI (blue). **C**, **D** Transwell migration assay and wound healing assay to evaluate the cell migration ability. Data are means ± SD, *P < 0.05, **P < 0.01, ***P < 0.001. One-way ANOVA followed by a Tukey’s test was used in (**A**–**D**).

As shown in Fig. 5A, enzyme-linked immunosorbent assay (ELISA) results revealed that TGF-β1 stimulation led to increased levels of TGF-β1 and inflammatory cytokines (TNF-α, IL-1β, and IL-6) in the cell culture supernatant, whereas CDA1 overexpression attenuated this effect. Next, 5-ethynyl-2’-deoxyuridine (EDU) staining showed an increased percentage of proliferating cells following stimulation with exogenous TGF‐β1 cytokine, which could be substantially abolished by the overexpression of the *Tspyl2* gene (Fig. 5B). Additionally, transwell migration and wound healing assays were conducted to identify the migration ability of HFL1 cells after infection with Lv-Tspyl2 (Fig. 5C, D). The results revealed a decreased migration rate in the Lv-Tspyl2 group compared to that in the control lentivirus (Lv-Control) group. TGF-β1 treatment dramatically promoted the migration of HFL1 cells; however, after CDA1 overexpression, the migration rate was significantly decreased in the Lv-Tspyl2+TGF-β1 group. These data suggest that CDA1 overexpression suppresses the lung fibroblast-to-myofibroblast transition, inflammation, cell proliferation and migration by abrogating TGF-β/Smad3 signaling in vitro.

### CDA1 knockdown promotes the lung fibroblast-to-myofibroblast transition and the release of inflammatory cytokines in vitro

**Fig. 6:**
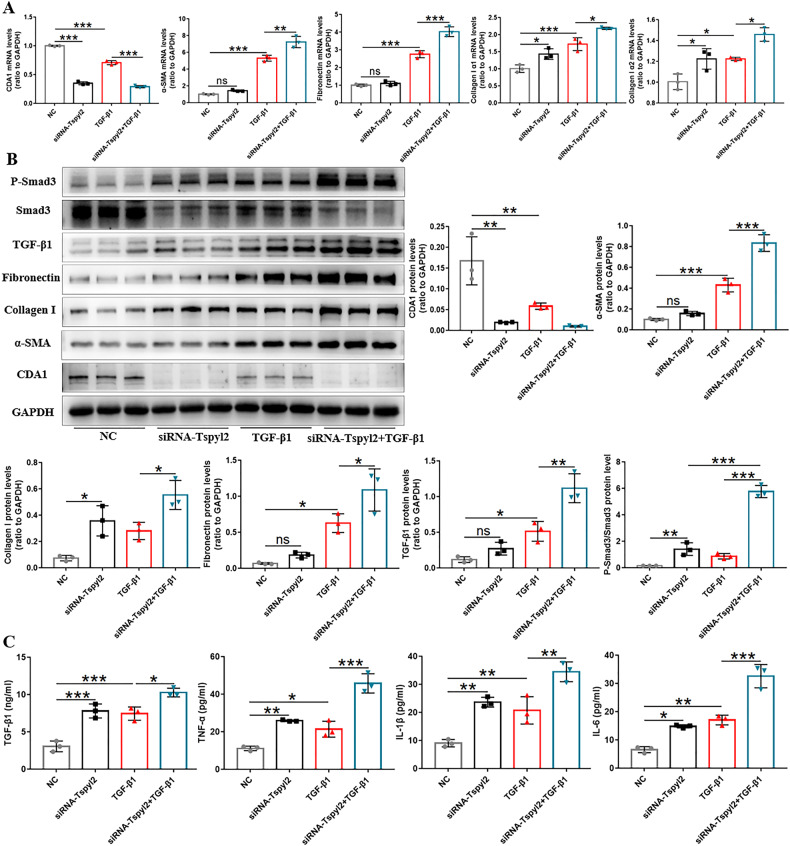
CDA1 knockdown promotes the lung fibroblast-to-myofibroblast transition, and increases the release of inflammatory cytokines in HFL1 cells. **A**, **B** Western blotting and qRT-PCR to test CDA1, fibrotic biomarkers, and TGF-β/Smad3 signaling at protein and mRNA levels after TGF-β1 or siRNA-Tspyl2 treatment. **C** Measurement of TGF-β1, TNF-α, IL-1β, and IL-6 in the cell culture supernatant by ELISA. Data are means ± SD, ns no significant difference, *P < 0.05, **P < 0.01, ***P < 0.001. One-way ANOVA followed by a Tukey’s test was used in (**A**–**C**). NC negative control group.

Furthermore, to verify the response of fibrotic biomarkers with a decrease in CDA1 protein level, the mRNA of CDA1 was degraded efficiently and specifically by small interfering RNA (siRNA)-Tspyl2 transfection, to construct a CDA1 knockdown HFL1 cell model. After 24 h of siRNA transfection, cells were stimulated with exogenous TGF‐β1 for 48 h. The mRNA and protein levels of CDA1 in the siRNA-Tspyl2 group were significantly lower than those in the negative control (NC) group. CDA1 knockdown further amplified the effect of TGF-β1-induced increases in α-SMA, collagen I, fibronectin, TGF-β, and P-Smad3/Smad3 expression in HFL1 cells (Fig. 6A, B). CDA1 knockdown further increased the release of TGF-β1, TNF-α, IL-1β, and IL-6 in the cell culture supernatant (Fig. 6C). These results confirmed the inhibitory effect of CDA1 on the fibroblast-to-myofibroblast transition, inflammation, and TGF-β/Smad3 signaling pathway in HFL1 cells.

### Preventive intratracheal delivery of AAV9-Tspyl2 retards BLM-induced PF by inhibiting the activation of TGF-β/Smad3 signaling

To assess the effect of *Tspyl2* gene therapy, we first assessed the gene transfer efficiency of adeno-associated virus serotype 9 (AAV9)-mediated gene transfer by detecting GFP protein and fluorescence signals in mouse lungs harvested 28 days after administration of control AAV9 vector encoding green fluorescent protein (GFP; AAV9-GFP). As shown in Supplementary Fig. 2A and B, we found a massive increase in GFP protein expression after intratracheal delivery of AAV9-GFP, and a viral load of 2.5×10^11^v. g./mice was sufficient. Under these conditions, GFP proteins were expressed abundantly in the endothelial cells of small pulmonary arteries, bronchial epithelial cells, and pneumocytes (Supplementary Fig. 2C). Unsatisfactorily, the protein expression of CDA1 in mouse lung tissues did not change significantly when AAV9-GFP was delivered via tail vein injection.

**Fig. 7:**
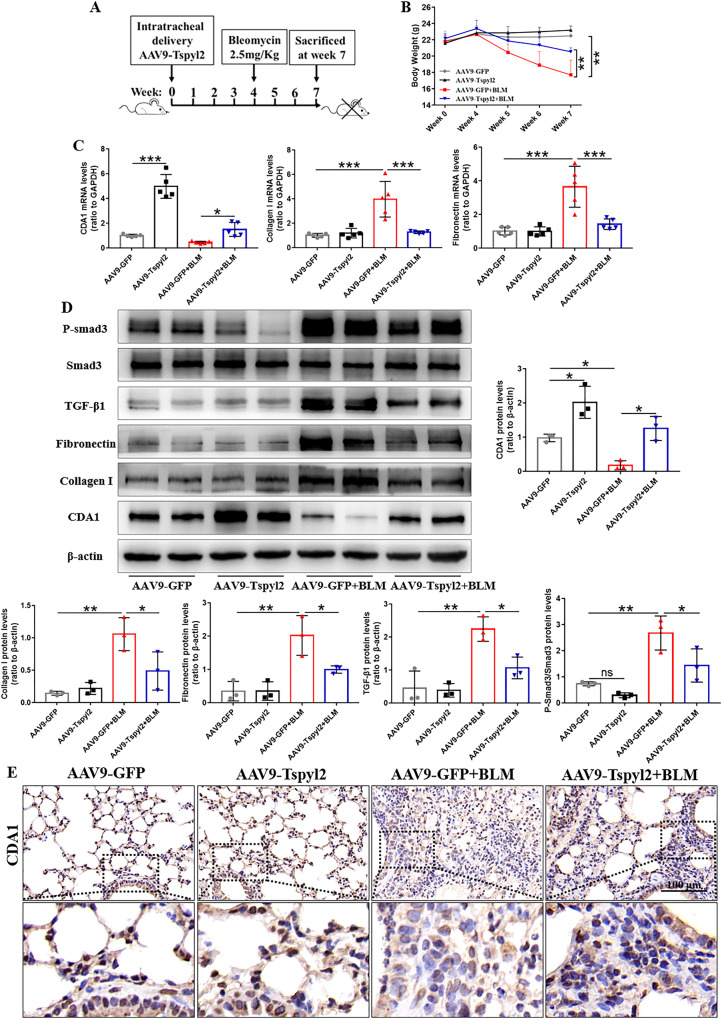
Preventive intratracheal delivery of AAV9-Tspyl2 retards the BLM-induced PF by inhibiting the activation of TGF-β/Smad3 signaling. **A** Schema of AAV9-Tspyl2 gene therapy in the BLM-induced PF model. **B** The mouse body weights at different time points throughout the experiment. **C**, **D** Western blotting and qRT-PCR to test CDA1, fibrotic biomarkers, and TGF-β/Smad3 signaling at protein and mRNA levels in mouse lung tissues. **E** Immunohistochemistry staining showing CDA1 expression (dark brown) in mouse lung tissues. Data are means ± SD, ns no significant difference, *P < 0.05, **P < 0.01, ***P < 0.001. One-way ANOVA followed by a Tukey’s test was used in (**B**–**D**).

Next, we evaluated the effect of the *Tspyl2* gene therapy. In the experimental protocol, mice were randomly allocated to a control AAV9-GFP group that received a single intratracheal aerosolization AAV9-GFP, and an AAV9 carrying the mouse Tspyl2 gene (AAV9-Tspyl2) group that received intratracheal AAV9-Tspyl2 injections. Four weeks later, all mice were randomly assigned to receive intratracheally aerosolized BLM or phosphate-buffered saline (PBS) solution (Fig. 7A). After a single intratracheal aerosolization of BLM, significant weight loss was observed in the AAV9-GFP + BLM group; however, CDA1 overexpression resulted in significant improvement of weight loss in AAV9-Tspyl2+BLM (Fig. 7B). Furthermore, quantitative real-time PCR (qRT-PCR), western blotting, and immunohistochemical staining showed that CDA1 was upregulated and expressed abundantly in the endothelial cells of small pulmonary arteries, bronchial epithelial cells, and pneumocytes in the lungs of AAV9-Tspyl2-treated mice, indicating successful AAV9-Tspyl2 delivery to the lungs (Fig. 7C, D, E). In the AAV9-GFP + BLM group, the mRNA and protein levels of collagen I and fibronectin increased significantly, whereas they were attenuated by AAV9-Tspyl2 treatment in the AAV9-Tspyl2+BLM group (Fig. 7C, D). Further analysis revealed that TGF-β1 protein level and P-Smad3/Smad3 protein ratio were upregulated in the BLM-induced PF model, while AAV9-Tspyl2 treatment reversed this effect (Fig. 7D).

**Fig. 8:**
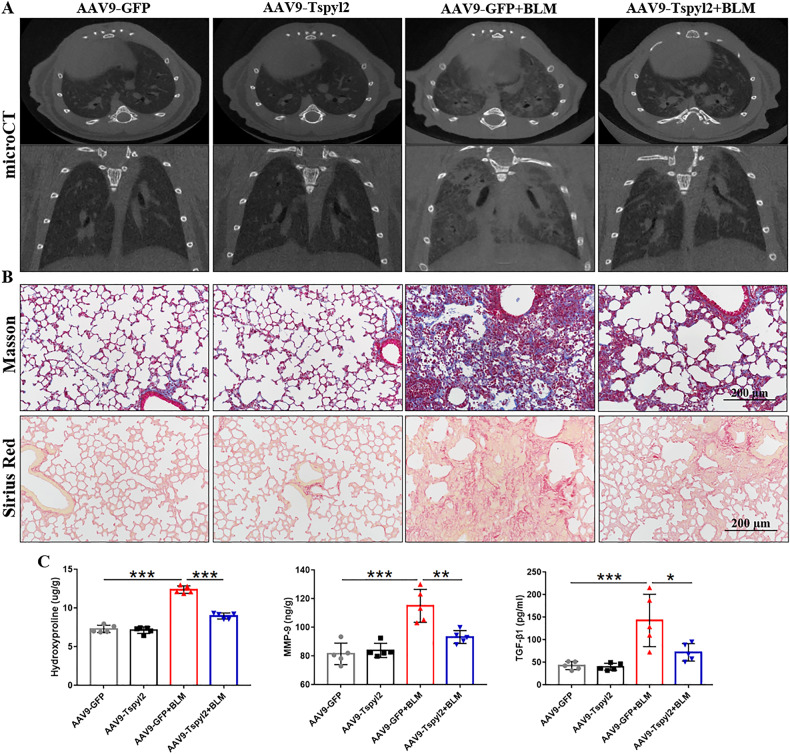
Preventive intratracheal delivery of AAV9-Tspyl2 attenuates lung fibrosis in the BLM-induced PF model. **A** Representative micro-CT images of chest in mice. **B** Masson and Sirius Red stained lung sections of mice. **C** Measurement of hydroxyproline and MMP-9 in the lung tissues and TGF-β1 in the BALF by ELISA. Data are means ± SD, ns no significant difference, *P < 0.05, **P < 0.01, ***P < 0.001. One-way ANOVA followed by a Tukey’s test was used in (**C**).

Microcomputed tomography (micro-CT) images revealed destroyed lung structures, multiple fiber strip shadows, and consolidation shadows in both lungs of the BLM-induced PF group, while in the AAV9-Tspyl2+BLM group, the structural destruction of lung tissues was alleviated (Fig. 8A). Masson and Sirus Red staining revealed abundant collagen deposition in the lung tissue after BLM treatment in mice. While, collagen deposition was significantly reduced in the AAV9-Tspyl2+BLM group compared to the AAV9-GFP + BLM group (Fig. 8B). ELISA analysis showed that AAV9-Tspyl2 treatment significantly decreased the concentrations of hydroxyproline and matrix metalloproteinase-9 (MMP-9) in the lung tissues and TGF-β1 in the bronchoalveolar lavage fluid (BALF) of mice in the BLM-induced PF model (Fig. 8C). There, CDA1 overexpression was verified to alleviate PF induced by BLM administration.

### CDA1 overexpression relieves BLM-induced inflammation in PF

**Fig. 9:**
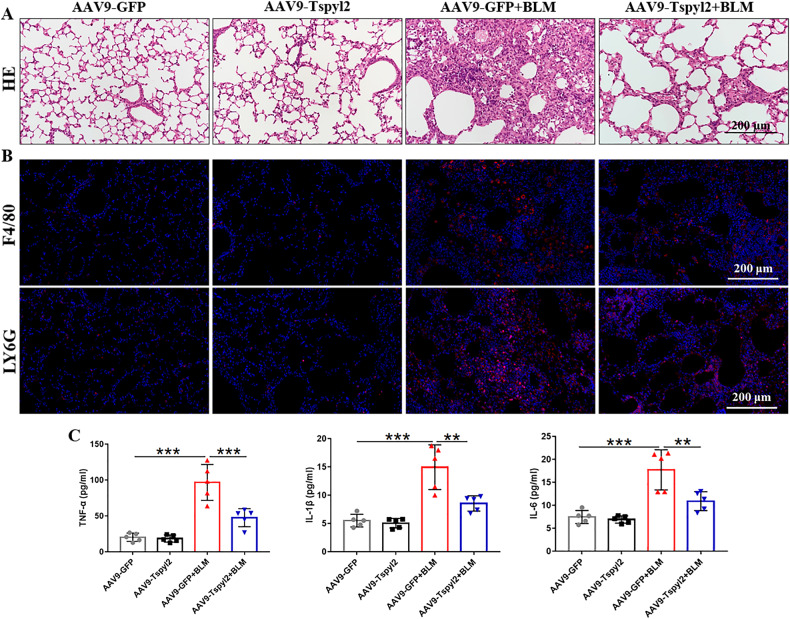
Preventive intratracheal delivery of AAV9-Tspyl2 attenuates inflammation in the BLM-induced PF model. **A** HE stained lung sections of mice. **B** Immunofluorescence staining showing Ly6G+ cells and F4/80+ macrophages in mouse lung tissues. **C** Measurement of TNF-α, IL-1β, and IL-6 in the BALF by ELISA. Data are means ± SD, **P < 0.01, ***P < 0.001. One-way ANOVA followed by a Tukey’s test was used in (**C**).

We further evaluated the inflammation in BLM-induced PF in mice. Hematoxylin-eosin (HE) staining showed a single intratracheal aerosolization of BLM resulted in obvious lung morphological changes, including thickening of the alveolar septum, destroyed alveolar structure, and heavy inflammatory cell infiltration in the alveolar space and lung interstitium; however, CDA1 overexpression reduced lung tissue damage (Fig. 9A). Immunofluorescence staining results also showed that F4/80+ macrophages and Ly6G+ neutrophils were significantly decreased in the AAV9-Tspyl2+BLM group (Fig. 9B). Furthermore, ELISA results indicated significant upregulation of TNF-α, IL-1β, and IL-6 expression in the BALF of AAV9-GFP + BLM group, whereas AAV9-Tspyl2 treatment suppressed the effect (Fig. 9C). These results suggest that CDA1 overexpression relieves BLM-induced inflammation in PF.

## Discussion

IPF is a devastating fibrotic lung disease with limited treatment options that seriously threatens the life and health of patients. The pathophysiology underlying PF is complex and elusive, and has not been fully elucidated. In this study, BLM aerosolization induced the production of pro-fibrotic and pro-inflammatory cytokines, mainly TGF-β1, TNF-α, IL-1β, and IL-6, and increased the expression of ECM proteins, such as α-SMA, collagen I, and fibronectin, in BLM-induced PF mice. Consistently, exogenous TGF‐β1 challenged lung fibroblasts produced more pro-fibrotic and pro-inflammatory cytokines and ECM proteins in vitro. We provided novel evidence that CDA1 overexpression through AAV9-Tspyl2 aerosolization in vivo or Lv-Tspyl2 infection in vitro exerted anti-inflammatory and antifibrotic effects, and CDA1 overexpression in human lung fibroblasts also inhibited cell proliferation and migration ability. Furthermore, we revealed that CDA1, as a transcription regulator, efficiently inhibited TGF-β1 signal transduction. These findings elucidate the molecular mechanisms by which CDA1 plays an antifibrotic role by inhibiting the lung fibroblast-to-myofibroblast transition and downstream TGF-β/Smad3 signaling pathway in BLM-induced PF.

BLM is a chemotherapeutic drug used to treat various malignant tumors, but a single aerosolization of high-dose BLM in mice led to early strong inflammation in the lung tissue, which transitioned into fibrosis after 5-7 days [22]. The BLM-induced PF model has histological features similar to those of IPF patients, including disruption of alveolar architecture, abnormal myofibroblast proliferation and transdifferentiation, and excessive deposition of ECM proteins, which mimics the nature of progressive fibrosis seen in IPF patients [23, 24]. Nonetheless, the fibrotic phase of BLM-induced PF is self-limiting and is ultimately reversible. Spontaneous regression of fibrosis, particularly in mice, typically occurs 3-4 weeks after the intratracheal administration of BLM [22, 25]. In our study, the time-course experiments revealed that the expression of CDA1 decreased on day 7 post-intratracheal BLM administration, coinciding with the increase in fibrotic biomarkers mainly on day 7. This was consistent with the previously reported transition time from inflammation to fibrosis. Moreover, CDA1 expression decreased mainly during the fibrotic phase, suggesting that CDA1 was closely related to PF.

The present study provides novel insights into the antifibrotic role of CDA1 in BLM-induced PF. However, these findings challenge several current studies on the pro-fibrotic effect of CDA1 in murine diabetic models of atherosclerosis and renal fibrosis [17–19]. Our data revealed that CDA1 expression decreased in human IPF, a mouse model of BLM-induced PF, and TGF‐β1 challenged lung fibroblasts. Conversely, Chai et al. found significantly elevated CDA1 expression in murine diabetic models of atherosclerosis and renal fibrosis, TGF-β-treated HK-2 cells, and vascular smooth muscle cells [17–19]. We speculate that the changes in CDA1 expression in response to TGF-β treatment may be inconsistent across cell types. Similarly, TGF-β treatment inhibits the proliferation of nearly all non-neoplastic epithelial, endothelial, hematopoietic, neural, and some types of mesenchymal cells but promotes the proliferation of other mesenchymal cells, such as fibroblasts and smooth muscle cells [26]. In addition, the pathogenesis of diabetes-related organ fibrosis and BLM-induced PF are significantly different. Diabetes-related organ fibrosis, which takes at least half a year, is the final pathological change in long-term chronic injury caused by multiple factors such as hyperglycemia, persistent inflammation, and dysfunction of vascular endothelial cells in diabetic animals [18, 19, 27]. Conversely, BLM-induced PF in mice, which takes only 2-3 weeks, originates from early strong inflammation in the lung tissue [22]. The severity of tissue damage in the PF is significantly greater than that in diabetes-related organ fibrosis. Further, no studies have reported the role of CDA1 in fibrosis of other organs, such as the liver and skin.

Additionally, IPF and lung cancer share many pathogenic similarities, including genetic and epigenetic markers [28]. The occurrence and development of tumors are also related to inflammatory cells, fibroblasts, and abnormally secreted ECM proteins [29–31]. Solid tumors are often accompanied by organ fibrosis and inflammatory cell infiltration [32, 33]. Likewise, the probability of tumorigenesis is greatly increased in fibrotic tissues [34, 35]. Numerous studies have reported that *Tspyl2* gene acts as a tumor suppressor gene, and its encoded protein CDA1 is significantly downregulated in various types of tumors, such as glioma, lung cancer, liver cancer, and prostate cancer [36–39]. To a certain extent, the tumor suppressor effect of CDA1 supports its antifibrotic role in BLM-induced PF. Notably, CDA1 can not only attenuate lung fibrosis but also further inhibit lung tumorigenesis in BLM-induced PF.

In other diseases, CDA1 can also maintain the normal development of the nervous system [40–43] and heart [44, 45], allowing them to function properly. In diabetes patients, CDA1 helps reduce the severity of aortic aneurysms [46]. CDA1 in host cells can also inhibit the growth and replication of Toxoplasma gondii [47, 48]. These results suggest that CDA1 plays a positive protective role in most diseases, which is consistent with its antifibrotic effect in BLM-induced PF.

Although the underlying pathophysiology of PF is elusive, all stages of PF is accompanied by inflammation [49]. Initially, it is an acute strong inflammation in lung tissues, which transitions into fibrosis with chronic inflammation after 5-7 days, in BLM-induced PF [22]. However, inflammation as an important etiology of IPF is controversial owing to the negative results of anti-inflammatory therapy [50–52]. Despite these concerns, our results revealed that CDA1 inhibited inflammation both in vivo and in vitro, suggesting that CDA1 may attenuate BLM-induced PF, partly by inhibiting the inflammation.

In our study, CDA1 also inhibited the proliferation and migration of HFL1 cells. Similarly, CDA1 inhibits the proliferation of HeLa cells [16], lung cancer cells (A549) [39], breast cancer cells (MCF-7) [39], and prostate cancer cells (LNCaP) [36], which is directly determined by its two cyclin-dependent kinase phosphorylation sites [16]. Concurrently, CDA1 can also inhibit the migration ability of A549 and MCF-7 cells [39]. The lung fibroblast-to-myofibroblast transition significantly enhanced the cell migration ability, which was a key factor for progressive PF; however, CDA1 overexpression could inhibit it. These results indicate that CDA1 is a novel target for PF retardation.

In summary, *Tspyl2* gene therapy attenuates BLM-induced PF, partly by inhibiting the lung fibroblast-to-myofibroblast transition and TGF-β/Smad3 signaling pathway. These data strongly suggest that CDA1 is an appropriate and promising molecular target with safety and effectiveness in blocking the pro-fibrotic effect of TGF-β, thus preventing, retarding, or treating PF.

## Materials and methods

### Microarray analysis from Gene Expression omnibus (GEO) datasets

To analyze the expression of CDA1 in the lung tissues of IPF patients, GSE53845 (comprising 40 IPF and 8 control samples) and GSE24206 (comprising 17 samples from 11 IPF patients [six patients provided a pair of samples from upper and lower lobes] and six control samples) microarray datasets were obtained from the GEO datasets (https://www.ncbi.nlm.nih.gov/). The mRNA differential expression of CDA1 in IPF patients and healthy controls was analyzed using the GEO2R online analysis tool.

### Animal models and treatments

Eight-week-old male specific-pathogen-free C57BL/6 mice (weight 21–25 g) were purchased from HuafuKang Biotechnology (Beijing, China). The intratracheal delivery of aerosolized BLM or AAV9-Tspyl2 was performed as previously described [53]. Briefly, the mice were anesthetized by isoflurane inhalation and secured in the supine position. A microsprayer tip was inserted into the trachea through the glottis, and 50 µL BLM (2.5 mg/kg), AAV9-Tspyl2 (2.5×10^11 ^v.g./mouse), or AAV9-GFP (2.5×10^11 ^v.g./mouse) was aerosolized evenly to the lungs. No sample-size estimation was performed to ensure adequate power to detect a pre-specified effect size. All animal experiments were strictly performed in accordance with the NIH Guide for the Care and Use of Laboratory Animals and were approved by the Committee on the Ethics of Animal Experiments of West China Hospital, Sichuan University (No.2020198 A).

In the first part of the experiment, mice received 2.5 mg/kg body weight BLM prepared in sterile PBS intratracheally and were randomly sacrificed by intraperitoneal injection of excessive pentobarbital sodium on days 3, 7, 14, 21, or 28 after BLM administration. As controls, mice were treated with an equal volume of PBS and sacrificed on day 0. In the second part, mice received AAV9 vectors intratracheally, followed by intratracheal delivery of BLM or PBS solution 4 weeks later. On this basis, 60 mice were randomly divided into four groups: mice were treated successively with AAV9-GFP and PBS solution (AAV9-GFP group), AAV9-Tspyl2 and PBS solution (AAV9-Tspyl2 group), AAV9-GFP and BLM (AAV9-GFP + BLM group), AAV9-Tspyl2 and BLM (AAV9-Tspyl2+BLM group). The body weights of the mice were measured weekly. No blinding was used throughout the experiment.

### Bronchoalveolar lavage fluid (BALF) collection

After occluding the left pulmonary hilus, the right lung was washed with 0.5 mL of iced PBS for three times, and a liquid recovery rate greater than 80% was considered qualified. The BALF need be centrifuged at 1000×g for 5 min, and the supernatant was stored at −80 °C for further analysis by ELISA.

### Transcriptome sequencing and bioinformatics analysis

Total RNA was isolated using Trizol (Invitrogen, USA) from control lung tissues and fibrotic lung tissues on day 21 after BLM administration. The total RNA was subjected to fragmentation, cDNA synthesis, adaptor ligation, and PCR enrichment. We performed paired-end sequencing using an Illumina Novaseq 6000 system (Illumina Corporation, San Diego, CA, USA). Raw data were then filtered using the Cutadapt tool, and the clean reads were further mapped to the mouse genome using TopHat. DEGs were screened using the edgeR package (|log_2_Fold change | >0.5, P < 0.05, false discovery rate [FDR] < 0.05). KEGG enrichment analysis was performed using the clusterProfiler R package.

### Microcomputed tomography (micro-CT) scanning of mice

As described previously [54], lung micro-CT was conducted in all mice under isoflurane anesthesia using a Quantum GX micro-CT scanner (PerkinElmer, Inc., Waltham, MA) on day 21 after BLM administration. The parameters of the X-ray tube were 90 kVp and 160 μA, and projection radiographs were obtained during the entire 360° gantry rotation, which took approximately 4.5 minutes.

### Histologic staining

Lung samples were formalin-fixed, paraffin-embedded, sectioned at 6 µm, and processed for routine HE staining, Masson staining, and Sirius Red staining for morphological analysis and localization of collagen expression using standard protocols. Immunohistochemical staining was performed on lung paraffin sections, which were rehydrated and labeled with primary antibodies against CDA1(1:100, Proteintech) at 4 °C overnight, and then labeled with peroxidase-conjugated secondary antibodies. Immunofluorescence staining was performed with a specific antibody against vimentin (1:100, Abcam), F4/80 (1:100, Abcam), and LY6G (1:100, Proteintech) at 4°C overnight and incubated with a secondary antibody. The nuclei were marked using 4′ 6-diamidino- 2-pheny-lindole (DAPI). Staining was quantified in five randomly microscopic fields on each slide.

### Western blotting

As reported previously [55], proteins in the lung or cells were extracted by RIPA lysis buffer containing protease and phosphatase inhibitors (Servicebio, China), and the protein concentration was determined using the BCA protein kit (Thermo, United States). After denaturation, proteins were separated by SDS-PAGE (Epizyme, China) and blotted onto activated PVDF membranes (Millipore, United States). After blocking with protein free rapid blocking buffer (Epizyme, China) for 10 min, the membranes were incubated with primary antibodies against GAPDH (1:50000, Proteintech), β-actin (1:50000, Proteintech), CDA1 (1:2000, Proteintech), α-SMA (1:1000, Abcam), collagen I (1:1000, Abcam), fibronectin (1:1000, Proteintech), TGF-β1 (1:1000, Proteintech), Smad3 (1:2000, Abcam), phosphorylated Smad3 (P-Smad3) (1:2000, Abcam), GFP (1:2000, Proteintech) at 4 °C overnight. After washing, the membranes were incubated with the appropriate secondary antibodies and visualized using an ECL Assay Kit (Epizyme, China). GAPDH/β-actin served as an internal control. The intensities of the target bands were quantified using ImageJ software.

### Quantitative real-time PCR (qRT-PCR)

Total RNA from lung tissues and cells was extracted using Trizol (Invitrogen, USA), and the RNA concentration was quantified using a NanoDrop 2000 Spectrophotometer. RNA reverse transcription was performed using the PrimeScript™ RT Reagent Kit (Takara, Japan) according to the manufacturer’s instructions. CDA1, α-SMA, collagen I, fibronectin, and TGF-β1 were amplified using SYBR Green Master Mix (Takara, Japan). Relative gene expression levels were normalized to those of GAPDH and β-actin and calculated using the 2^−ΔΔCt^ method. The primer sequences are listed in Table 1.

**Table 1:** Sequences of primers used for qRT-PCR.

Gene	Primer	Sequence (5’ → 3’)
Mus-GAPDH	Forward	CCTCGTCCCGTAGACAAAATG
	Reverse	TGAGGTCAATGAAGGGGTCGT
Mus-β-actin	Forward	GGCTGTATTCCCCTCCATCG
	Reverse	CCAGTTGGTAACAATGCCATGT
Mus-Tspyl2	Forward	TGACCCAGAGGAAAAGAATACC
	Reverse	CACTGCTGTCCTGGATATTAGT
Mus-α-SMA	Forward	GTCCCAGACATCAGGGAGTAA
	Reverse	TCGGATACTTCAGCGTCAGGA
Mus-Collagen I	Forward	AAGAAGCACGTCTGGTTTGGAG
	Reverse	GGTCCATGTAGGCTACGCTGTT
Mus-TGF-β	Forward	CAACAATTCCTGGCGTTACCTT
	Reverse	TCGAAAGCCCTGTATTCCGTCT
Mus-Fibronectin	Forward	GCTCAGCAAATCGTGCAGC
	Reverse	CTAGGTAGGTCCGTTCCCACT
Homo-GAPDH	Forward	GGTGGTCTCCTCTGACTTCAACA
	Reverse	TCTCTTCCTCTTGTGCTCTTGCT
Homo-Tspyl2	Forward	AACAACAACGAGAACACTTACG
	Reverse	TATCTTCATCATCACTGGCCTC
Homo-α-SMA	Forward	TCGTGCTGGACTCTGGAGATGG
	Reverse	CCACGCTCAGTCAGGATCTTCATG
Homo-Collagen I α1	Forward	AAAGATGGACTCAACGGTCTC
	Reverse	CATCGTGAGCCTTCTCTTGAG
Homo-Collagen I α2	Forward	CTCCATGGTGAGTTTGGTCTC
	Reverse	CTTCCAATAGGACCAGTAGGAC
Homo-Fibronectin	Forward	GGCGACAGGACGGACATCTTTG
	Reverse	GGCACAAGGCACCATTGGAATTTC

### Chromatin immunoprecipitation (ChIP) assays

The ChIP assays was conducted using a ChIP Kit (BaiweiBio, China). In brief, 3×10^7^ HFL1 cells were collected, cross-linked with 1% formaldehyde, terminated with glycine, and sonicated to an average DNA length of 200 bp~600 bp. The sheared chromatin was immunoprecipitated with anti-IgG (Proteintech, China) or anti-CDA1 (Proteintech, China) antibody beads. Then, the protein-chromatin complexes were eluted off the beads, and the cross-linking was reversed. DNA fragments were purified by phenol: chloroform: isoamyl alcohol and precipitated by anhydrous ethanol. The immunoprecipitated DNA was quantified by real-time PCR using SYBR Green Master Mix (Thermo, United States). The transcription start site of TGF-β1 to the upstream 2000bp was defined as the promoter region, which was amplified with 9 pairs of primers. Primers used for this study are summarized in Supplementary Table 1. The percentage of input was calculated using 2^−ΔCt(normalized ChIP)^. ΔCt(normalized ChIP) = Ct(ChIP) – (Ct [input] − log_2_[input dilution factor]).

### Enzyme-linked immunosorbent assay (ELISA)

The contents of hydroxyproline and MMP-9 in lung tissues and the levels of TGF-β1, TNF-α, IL-6, and IL-1β in BALF and cell culture supernatant were detected by ELISA kits assaying (Ruixinbio, China) following the manufacturer’s instructions, respectively.

### Cell culture and TGF-β1 treatment

HFL1 cells were purchased from the National Collection of Authenticated Cell Cultures and cultured in Ham’s F-12K medium (Gibco, USA) containing 10% fetal bovine serum (FBS; Gibco, USA), 1% GlutaMAX (Gibco, USA), 1% non-essential amino acids (Gibco, USA), and 1% pyruvate (Gibco, USA).

Primary lung fibroblasts were isolated from the mouse lung, purified, and identified as previously described [54]. Briefly, the lungs from suckling mice (one-week-old) were collected, washed well to remove blood, minced into small pieces (1×1×1 mm), and seeded in Dulbecco’s modified Eagle’s medium containing 10% FBS (Gibco, USA). After 3 days, spindle-shaped fibroblasts started to crawl out of the tissue mass, resulting in approximately 80% confluence in 1 week. At this point, the lung primary fibroblasts were purified and identified by vimentin immunofluorescence, and the results revealed a fibroblast purity of nearly 100%. Purified cells were cultured and used for subsequent processing.

All cells were grown at 37 °C in a humidified atmosphere containing 5% CO_2_. HFL1s or lung primary fibroblasts were stimulated in vitro with TGF-β1 (Peprotech, USA) for 24-72 h.

### siRNA experiments

Human siRNA-Tspyl2 and negative control siRNA were purchased from Tsingke Biotechnology (Beijing, China). HFL1 cells were seeded at approximately 2×10^5^ cells/well in 12-well plates and maintained in at 37 °C incubator until the cells reached approximately 50% confluence before transfection. Transient transfection with 50 nM siRNAs in each well was performed using Lipofectamine 3000 (Invitrogen, USA), according to the manufacturer’s instructions. The knockdown effect was verified using western blotting and qRT-PCR after 72 h. When TGF-β treatment was required, the cells were washed with PBS 24 h after siRNA transfection and incubated with fresh culture medium containing TGF-β1 (10 ng/mL) for 48 h.

### Lentivirus experiments

The Lv-Tspyl2 and Lv-Control was purchased from GeneChem, Inc. (Shanghai, China). Recombinant *Tspyl2* lentiviruses were transduced into HFL1 cells at a multiplicity of infection of 10 with a virus infection enhancer following the manufacturer’s instructions. Twelve hours later, the old medium containing lentiviruses was removed and replaced with fresh medium. After another 72 h, the transduced cells were selected using 2 µg/mL puromycin (GeneChem, China) to obtain cells with stable overexpression of CDA1.

### Cell proliferation

The proliferation of HFL1s was measured by EDU incorporation for 6 h using the BeyoClick™ EDU Cell Proliferation Kit with Alexa Fluor 488 (Beyotime Biotechnology, China) according to the manufacturer’s instructions. Briefly, cultured HFL1 cells were infected with Lv-Control or Lv-Tspyl2 for at least 72 h, after which infected cells were seeded in 24-well plates and treated with TGF-β for 48 h. During the last 6 h, HFL1 cells were incubated with 10 μM EDU staining buffer. Next, the cells were fixed, permeabilized, incubated with the click reaction mixture, and stained with DAPI.

### Transwell migration assay

Approximately 3×10^4^ lentivirus-infected HFL-1 cells (0.1 mL) were cultured in the upper transwell chamber (8-μm pore diameter) (Corning, United States) in serum-free medium. 0.6 ml culture solution with TGF-β1 (10 ng/mL) and 10% FBS was added to the lower cavity, and the control group was only treated with FBS. After 24 h of culture, the cells in the chamber were wiped off. Cells that passed outside the chamber were stained with crystal violet and subsequently photographed under a microscope.

### Wound healing assay

The lentivirus-infected HFL-1 cells were seeded at 5×10^4^ cells/well (70 µL) into an Ibidi Culture-Insert 2 Well (Ibidi, Germany) and allowed to grow for 24 h resulted in a confluent layer. Then, a cell-free gap of 500 µm was created after removing the culture-insert, and the cells were washed with PBS to remove non-adherent cells. We then provided fresh medium containing TGF-β1 (10 ng/mL) and photographed the plate at 0 and 24 h to capture two different fields at each time point on each plate. The number of cells that migrated into the wound space was manually counted in three fields per well.

### Statistical analyses

Statistical analysis was performed using the GraphPad Prism 7.0 software (GraphPad Software, USA). All inclusion/exclusion criteria were pre-established, and no animals or samples were excluded in the analysis. Results were presented as means ± standard deviations (SD) from at least three separate experiments. Raw data were analyzed using the student’s t test (two-tailed) for comparisons between two groups or one-way analysis of variance (ANOVA) with Tukey’s correction for comparisons among ≥3 groups. Differences were considered statistically significant at p < 0.05.

## Supplementary information

Supplementary Table 1

supplementary figure legends

Supplementary Figure 1

Supplementary Figure 2

aj-checklist

cdd-author-contribution-form

the full length uncropped original western blots

## Data Availability

All data that support the findings of this study are available from the corresponding author upon reasonable request.
