# Supplementary material for: AAV9-Tspyl2 gene therapy retards bleomycin-induced pulmonary fibrosis by modulating downstream TGF-β signaling in mice
Source: Cell Death Dis. 2023 Jun 30;14(6):389. doi: 10.1038/s41419-023-05889-8 (PMC10313802; doi:10.1038/s41419-023-05889-8)
Supplement: Supplementary file 1 — Supplementary Table 1 [file 41419_2023_5889_MOESM1_ESM.docx]

Supplementary Table 1. Sequences of primers used for CHIP.

| TGF-β1 promotor primer | Primer | Sequence (5'→3') | Gene length |
| --- | --- | --- | --- |
| TGF-β1 promotor primer 1 | Forward | ACATGGCCTACTCCCTTCCT | 239bp |
|  | Reverse | CATGAACATGGATGGCAGAC |  |
| TGF-β1 promotor primer 2 | Forward | CTGCCATCCATGTTCATGTC | 161bp |
|  | Reverse | CATAGCTTTGCCCATGTCCT |  |
| TGF-β1 promotor primer 3 | Forward | TGGATTAGAGAGGGGCAAGA | 241bp |
|  | Reverse | CCCCAGTGCCCTCAGTATAA |  |
| TGF-β1 promotor primer 4 | Forward | AGAAGGGCCTTGAATGTTGA | 202bp |
|  | Reverse | GGCCCAGTCTTTTCCTCTCT |  |
| TGF-β1 promotor primer 5 | Forward | CCTGGGGTCTCCAGTGAGTA | 175bp |
|  | Reverse | ACATGGCAAAATCCATAGCC |  |
| TGF-β1 promotor primer 6 | Forward | GGCTATGGATTTTGCCATGT | 260bp |
|  | Reverse | AGAGTCCCTCAGCACTCCAC |  |
| TGF-β1 promotor primer 7 | Forward | GTGGAGTGCTGAGGGACTCT | 168bp |
|  | Reverse | GACCCCGAGGTCCTAGAAAG |  |
| TGF-β1 promotor primer 8 | Forward | CCATCCTTCAGGTGTCCTGT | 199bp |
|  | Reverse | TGGGTCACCAGAGAAAGAGG |  |
| TGF-β1 promotor primer 9 | Forward | CACAGCGCATCTGGATCAC | 152bp |
|  | Reverse | GGCCACCGTCCTCATCTC |  |
| GAPDH | Forward | AAAAGCGGGGAGAAAGTAGG | 212bp |
|  | Reverse | AAGAAGATGCGGCTGACTGT |  |
