# Supplementary material for: AAV9-Tspyl2 gene therapy retards bleomycin-induced pulmonary fibrosis by modulating downstream TGF-β signaling in mice
Source: Cell Death Dis. 2023 Jun 30;14(6):389. doi: 10.1038/s41419-023-05889-8 (PMC10313802; doi:10.1038/s41419-023-05889-8)
Supplement: Supplementary file 2 — supplementary figure legends [file 41419_2023_5889_MOESM2_ESM.docx]

**Supplementary Figure Legends**

**Supplementary Figure 1.** The BLM-induced PF model in mice was established successfully. A. Time-course analysis of fibrotic biomarkers using qRT-PCR analysis in the lung tissues with BLM-induced PF in mice. B. Masson and Sirius Red stained lung sections of mice. The fibrotic green area in Masson staining and red area in Sirius Red staining indicated extracellular fibrotic collagen deposition. Data are means ± SD, ns: no significant difference, *P<0.05, **P<0.01, ***P<0.001. Student’s t test was used in (A, B), and each group was compared with the control group.

**Supplementary Figure 2.** Efficient intratracheal delivery of AAV9 vectors in mice. A and B. Western blotting analysis of GFP protein in the lungs of mice 4 weeks after the administration of AAV9-GFP. C. Representative images of GFP auto-fluorescence (green) and DAPI (Blue) in the lungs of mice 4 weeks after a single intratracheal aerosolization of AAV9-GFP at a dose of 2.5×10^11^ v.g./mice. Data are means ± SD; ns: no significant difference, **P<0.01. Student’s t test was used in (B), and each group was compared with the control group. IT, intratracheal delivery group. Vein, tail vein injection group.
