# Supplementary figures and images for: AAV9-Tspyl2 gene therapy retards bleomycin-induced pulmonary fibrosis by modulating downstream TGF-β signaling in mice
Source: Cell Death Dis. 2023 Jun 30;14(6):389. doi: 10.1038/s41419-023-05889-8 (PMC10313802; doi:10.1038/s41419-023-05889-8)

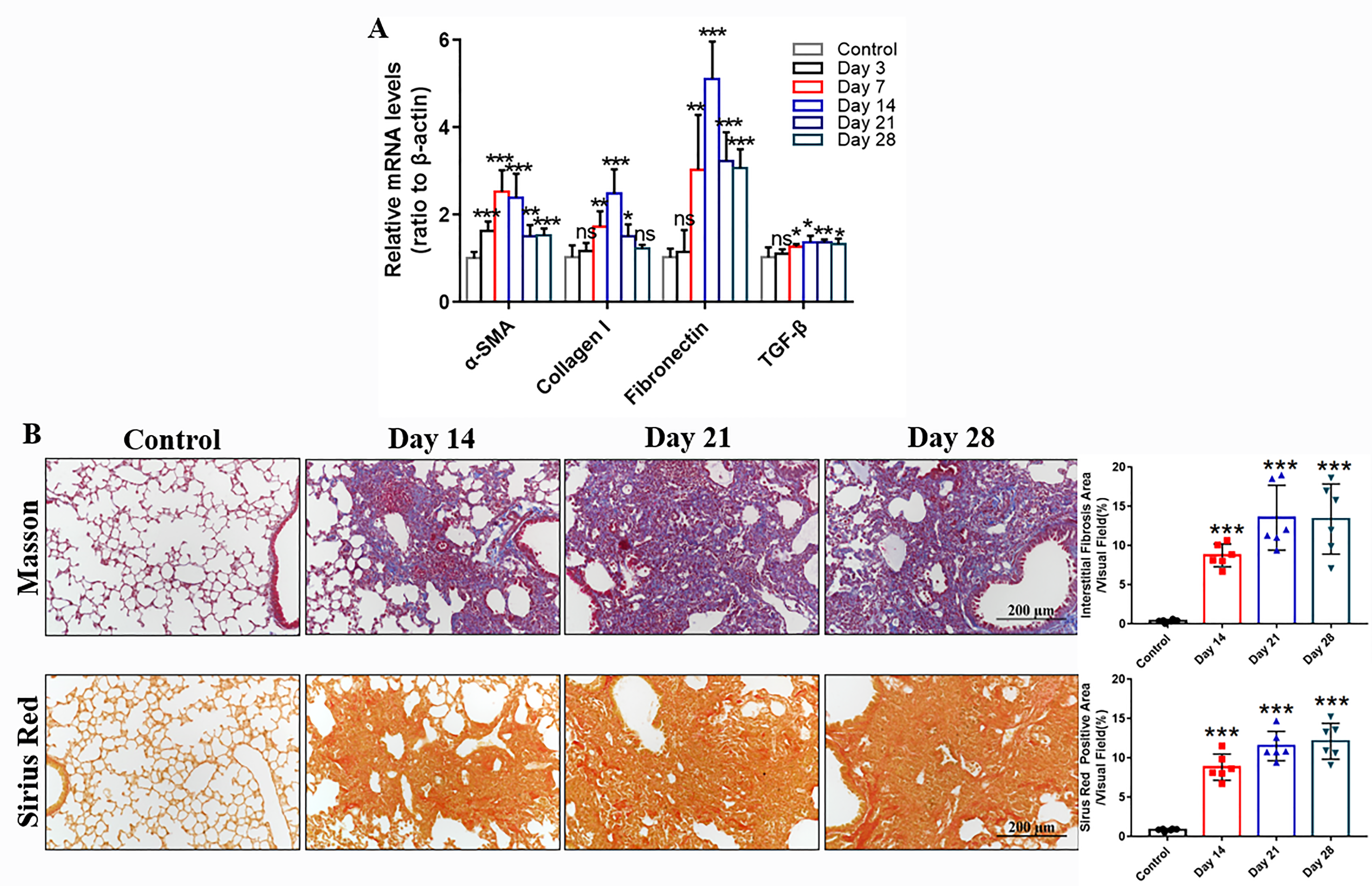

Supplement: Supplementary file 3 — Supplementary Figure 1 [file 41419_2023_5889_MOESM3_ESM.tif]

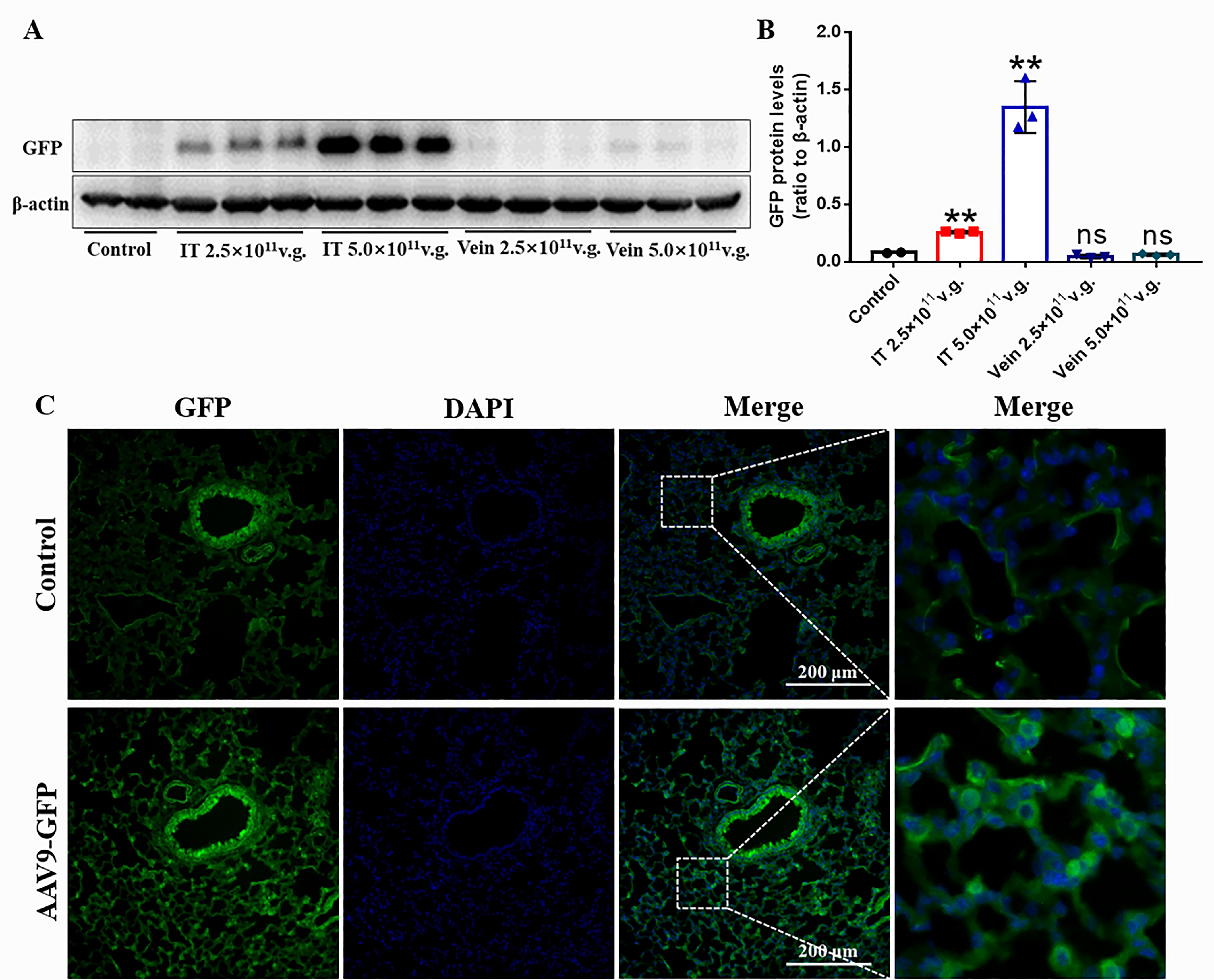

Supplement: Supplementary file 4 — Supplementary Figure 2 [file 41419_2023_5889_MOESM4_ESM.tif]
