# Supplementary material for: AAV9-Tspyl2 gene therapy retards bleomycin-induced pulmonary fibrosis by modulating downstream TGF-β signaling in mice
Source: Cell Death Dis. 2023 Jun 30;14(6):389. doi: 10.1038/s41419-023-05889-8 (PMC10313802; doi:10.1038/s41419-023-05889-8)
Supplement: Supplementary file 6 — cdd-author-contribution-form [file 41419_2023_5889_MOESM6_ESM.pdf]

DECLARATION OF CONTRIBUTIONS TO ARTICLE **ADMC**

Manuscript Number: CDDIS-22-4563-T

Journal Name: Cell *Death & Disease* (the 'Journal')

Proposed Title of the Contribution:

AAV9-Tspyl2 gene therapy retards bleomycin-induced pulmonary fibrosis by modulating downstream TGF-β signaling in mice (the 'Contribution)

Author(s):

Shijie Zhang, Xiang Tong, Sitong Liu, Jizhen Huang, Li Zhang, Tianli Zhang, Dongguang Wang, Hong Fan (the 'Authors')

For all *CDD* articles, each person named as an author in the published version must be able to show he or she has contributed substantially to the article.

Authorship credit should be based on 1) substantial contributions to conception and design, acquisition of data, or analysis and interpretation of data; 2) drafting the article or revising it critically for important intellectual content; and 3) final approval of the version to be published. Authors should meet conditions 1, 2 and 3.

Any person who cannot be shown to have made a substantial contribution to the article cannot be listed as an author in the final version. The name of any person who is deemed to have made a minor contribution can, however, appear in the Acknowledgments section of the article.

Please complete the table below to indicate the contributions of all named authors to the manuscript.

Author Full Name:	Specification of Contribution to the Manuscript:
Shijie Zhang	conception and design, analysis and interpretation of data, conducting experiments, drafting the manuscript, and final approval of the article.
Xiang Tong	conception and design, analysis and interpretation of data, conducting experiments, revising the article, and final approval of the article.
Sitong Liu	analysis and interpretation of data, conducting experiments, revising the article, and final approval of the article.
Jizhen Huang	conducting experiments, revising the article, and final approval of the article.
Li Zhang	conducting experiments, revising the article, and final approval of the article.
Tianli Zhang	conducting experiments, revising the article, and final approval of the article.
Dongguang Wang	conducting experiments, revising the article, and final approval of the article.
Hong Fan	conception and design, revising the article, and final approval of the article.

Please complete the table below to indicate the contributions of all named authors to the figures.

Figure 1:

SZ, XT, TZ and DW generated the data, prepared panel A-I, and assembled the figure.

Figure 2:

SZ, XT, SL, JH and LZ generated the data, prepared panel A-I, and assembled the figure.

Figure 3:

SZ, XT, SL, JH and LZ generated the data, prepared panel A-I, and assembled the figure.

Figure 4:

SZ, XT, SL, JH and LZ generated the data, prepared panel A-I, and assembled the figure.

Figure 5:

SZ, XT, SL, JH and LZ generated the data, prepared panel A-I, and assembled the figure.

Figure 6:

SZ, XT, TZ and DW generated the data, prepared panel A-I, and assembled the figure.

Signed for and on behalf of the Author(s):	Print Name:	Date:
Hong Fan	Hong Fan	November 4, 2022
