# Supplementary material for: AAV9-Tspyl2 gene therapy retards bleomycin-induced pulmonary fibrosis by modulating downstream TGF-β signaling in mice
Source: Cell Death Dis. 2023 Jun 30;14(6):389. doi: 10.1038/s41419-023-05889-8 (PMC10313802; doi:10.1038/s41419-023-05889-8)

Figure 1D-CDA1-1

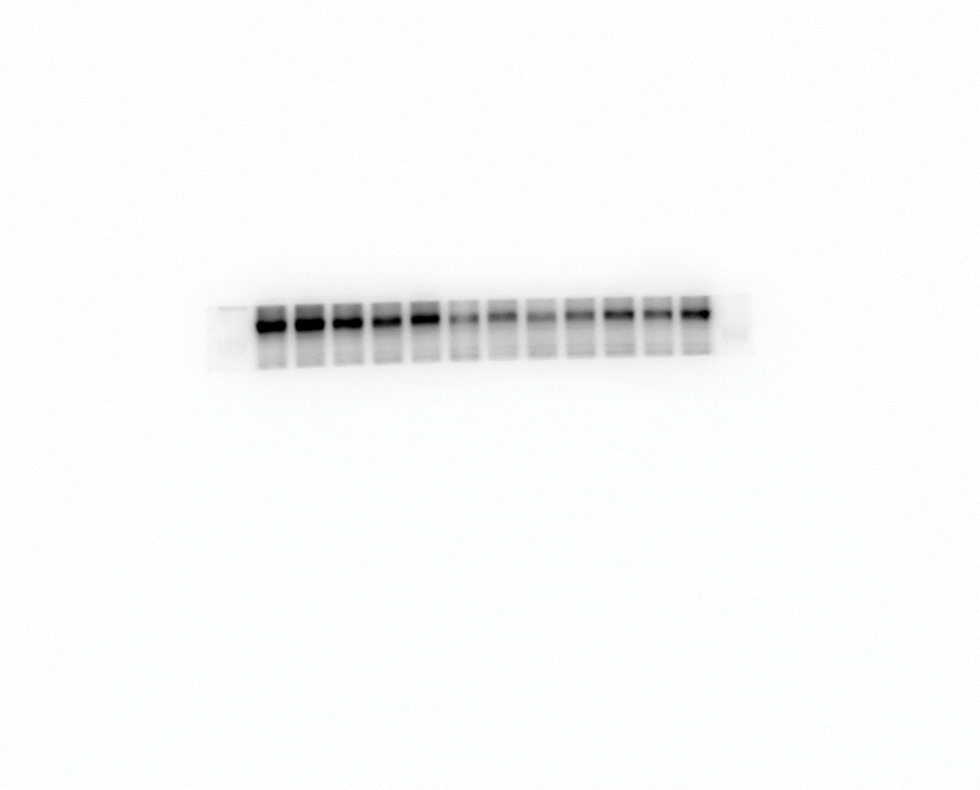

Figure 1D-CDA1-2

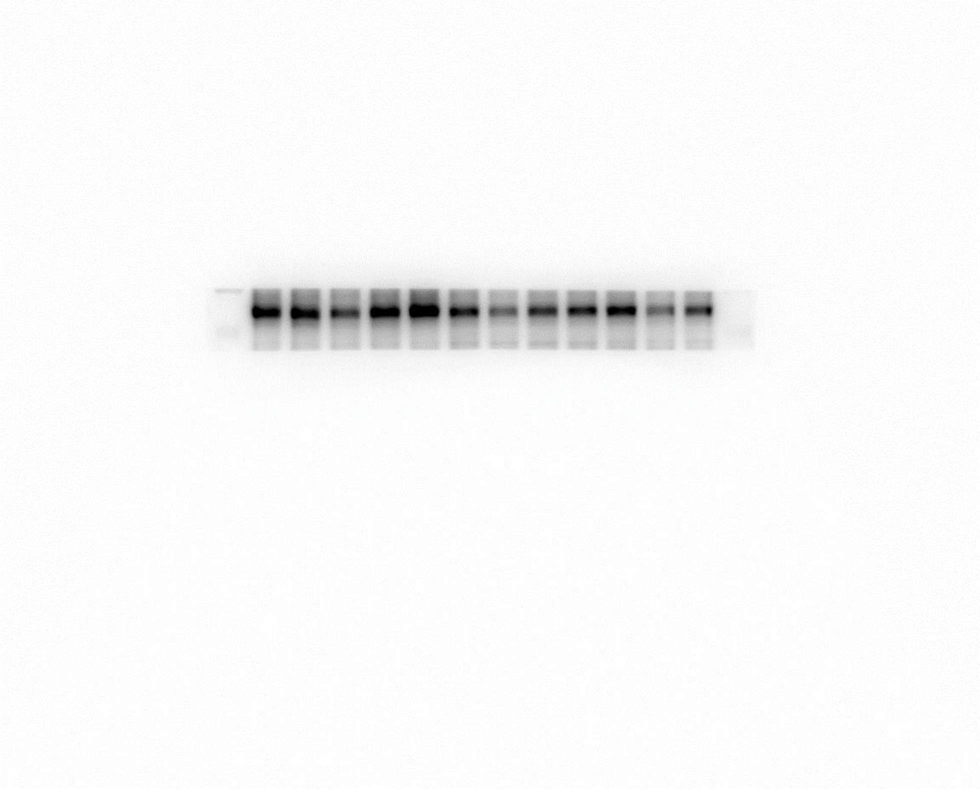

Figure 1D-CDA1-3

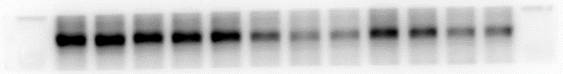

Figure 1D-CDA1-4

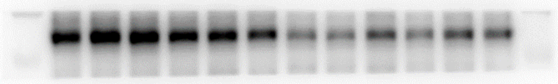

Figure 1D-GAPDH-1

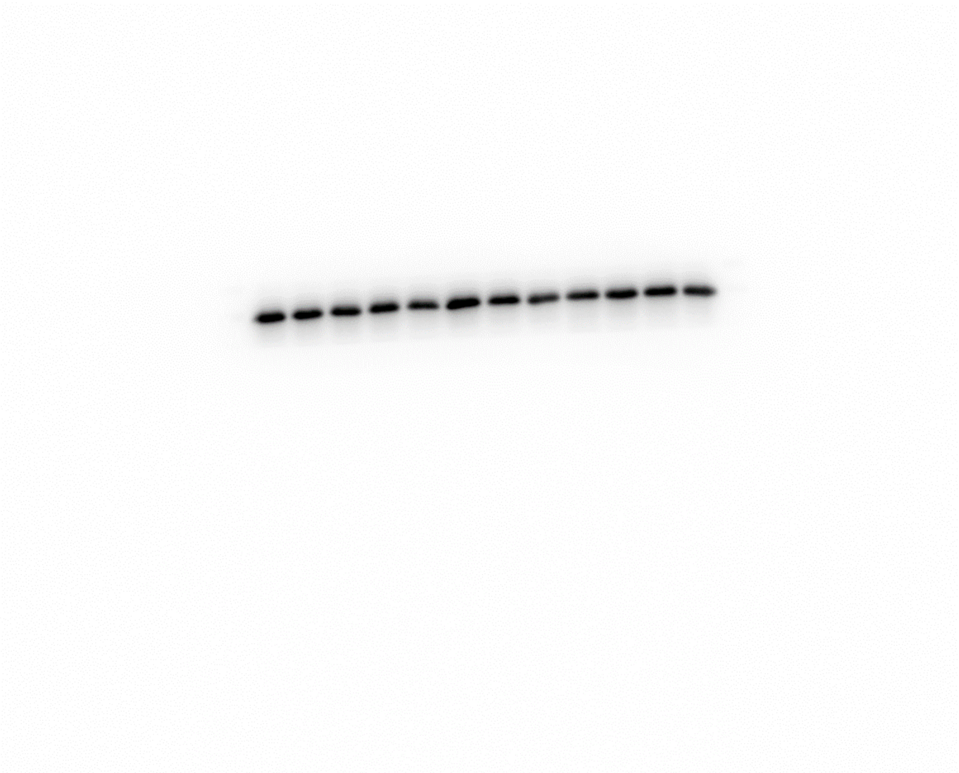

Figure 1D-GAPDH-2

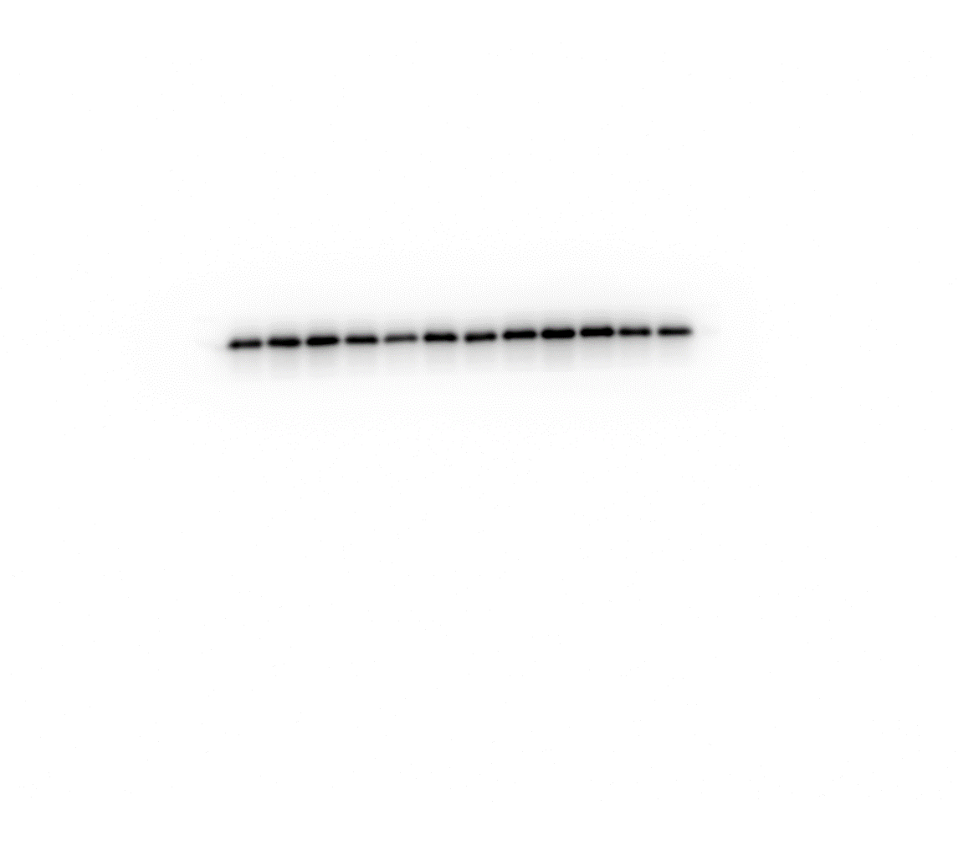

Figure 1D-GAPDH-3

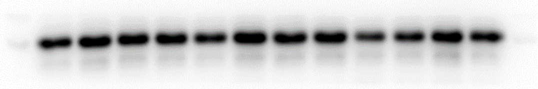

Figure 1D-GAPDH-4

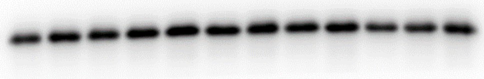

Figure 2A-a-SMA

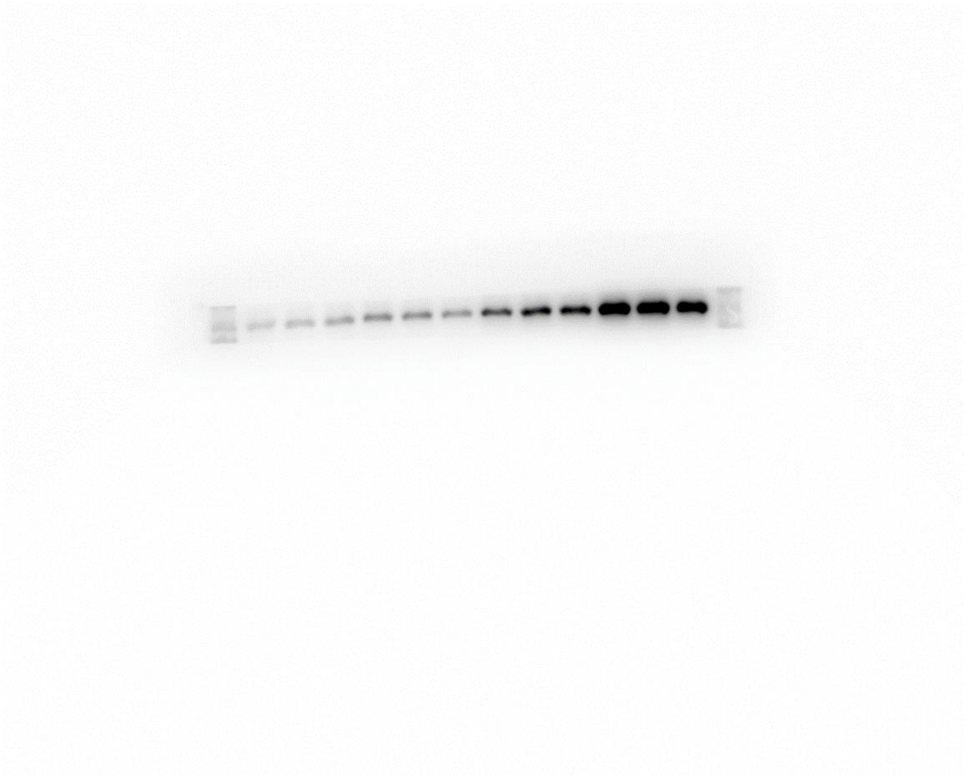

Figure 2A-CDA1

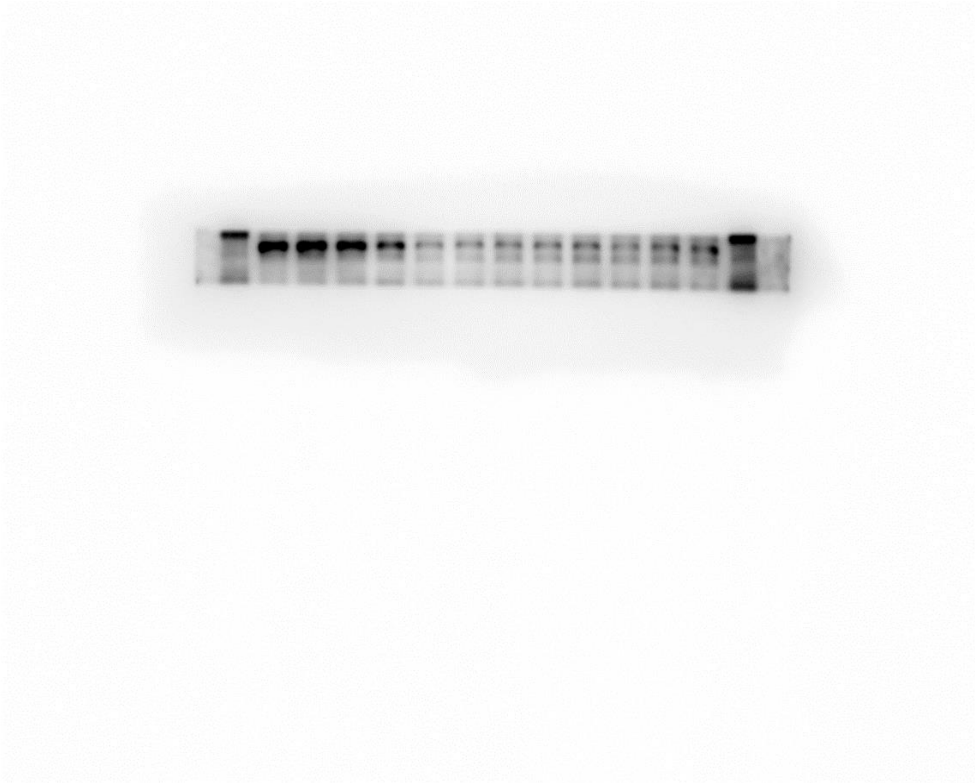

Figure 2A-collagen I

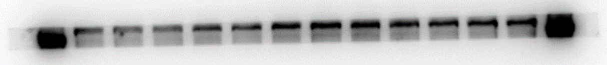

Figure 2A-Fibronectin

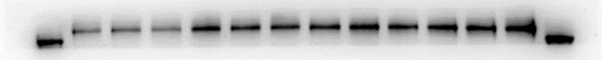

Figure 2A-GAPDH

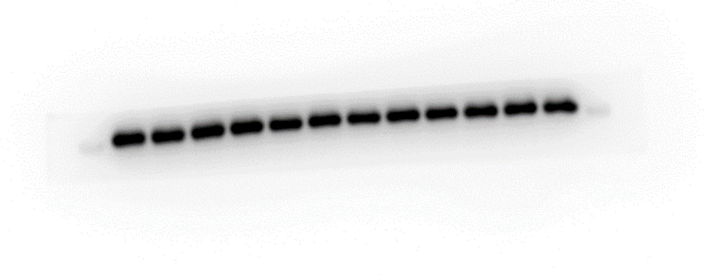

Figure 2D-a-SMA

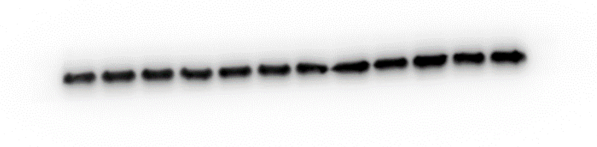

Figure 2D-CDA1

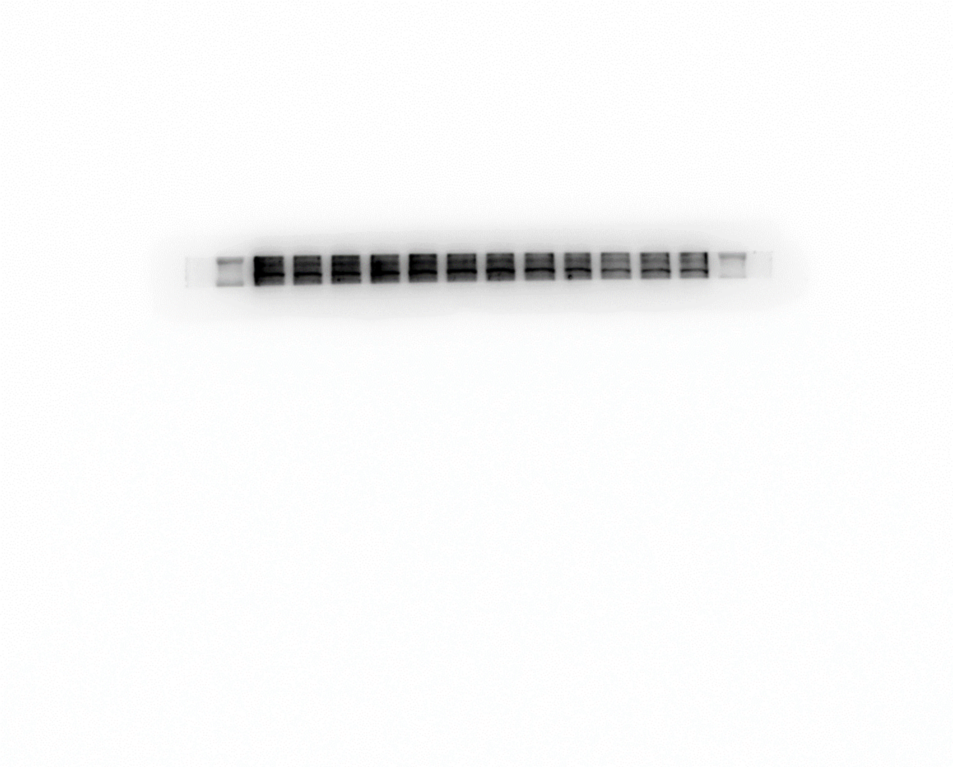

Figure 2D-Collagen I

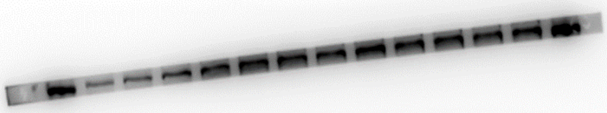

Figure 2D-Fibronectin

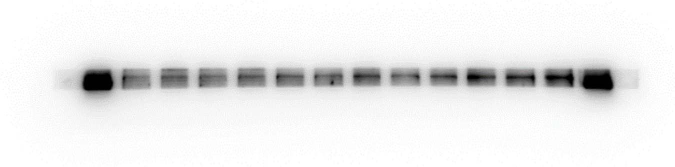

Figure 2D-β-actin

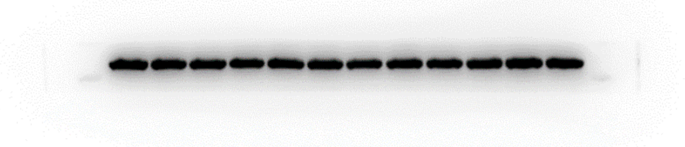

Figure 3A-a-SMA

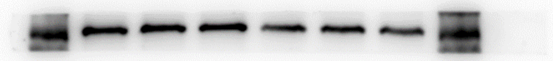

Figure 3A-CDA1

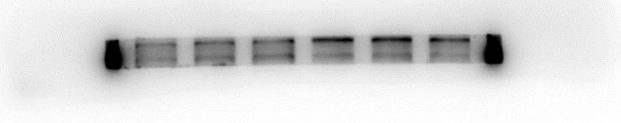

Figure 3A-Collagen I

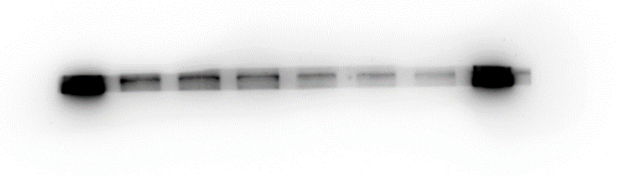

Figure 3A-Fibronectin

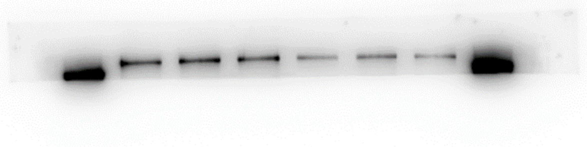

Figure 3A-GAPDH

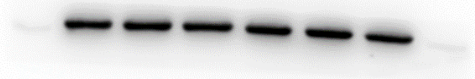

Figure 3A-TGF-β

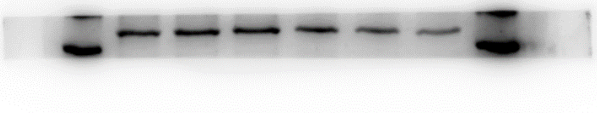

Figure 3C-a-SMA-1

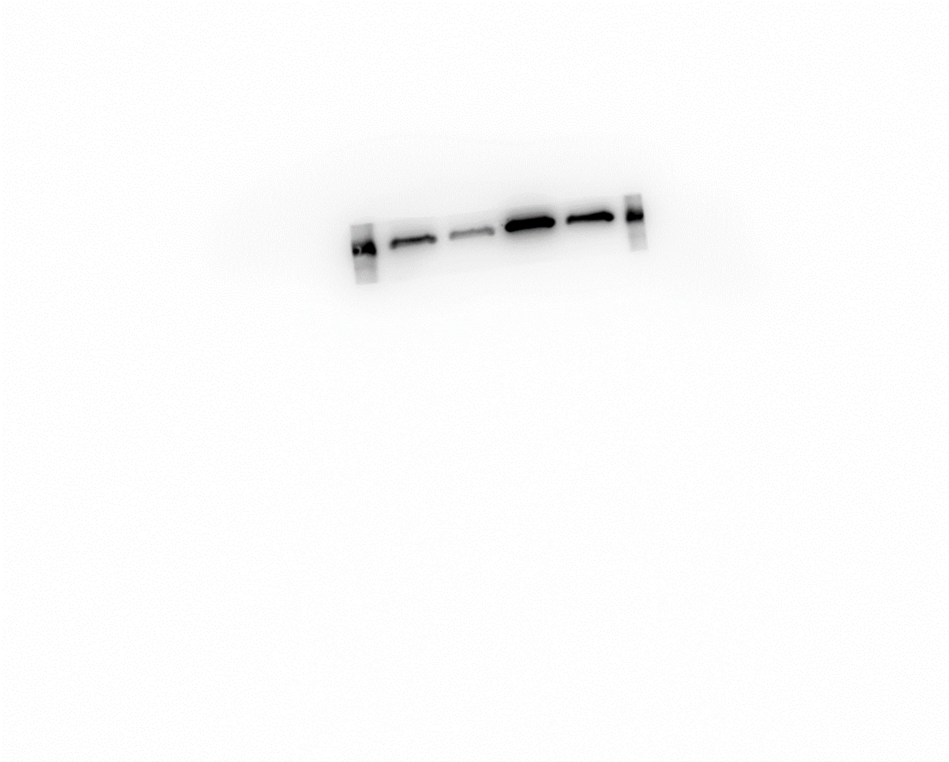

Figure 3C-a-SMA-2

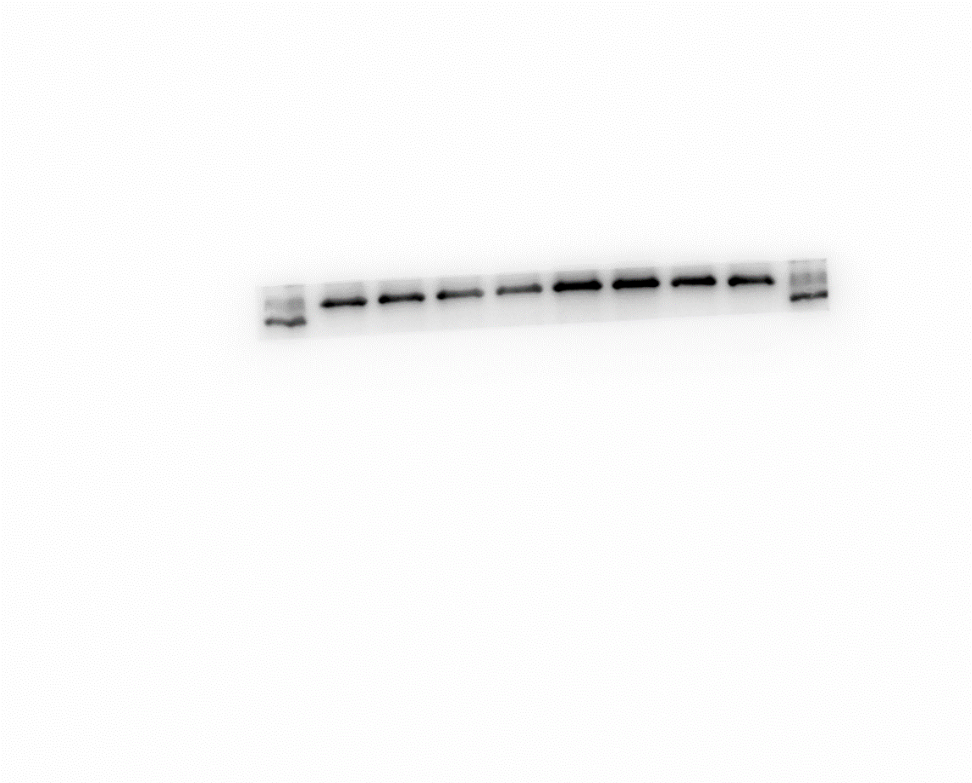

Figure 3C-CDA1-1

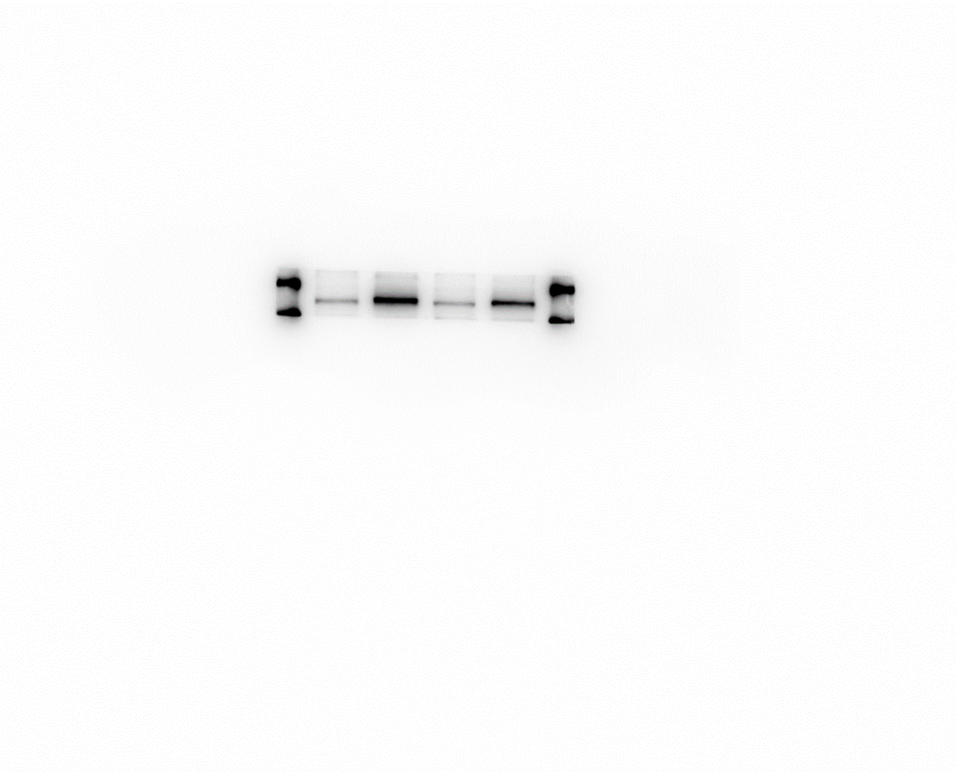

Figure 3C-CDA1-2

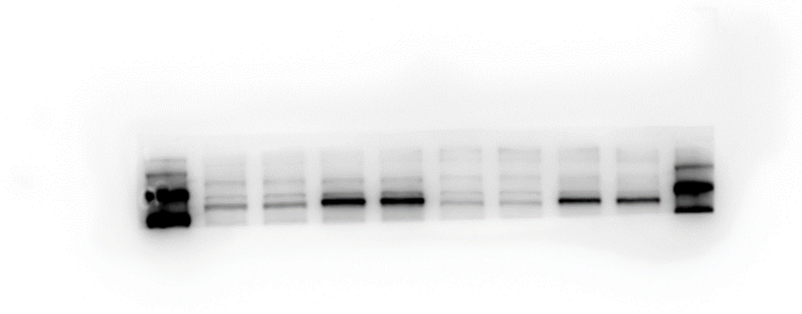

Figure 3C-Collagen-I-1

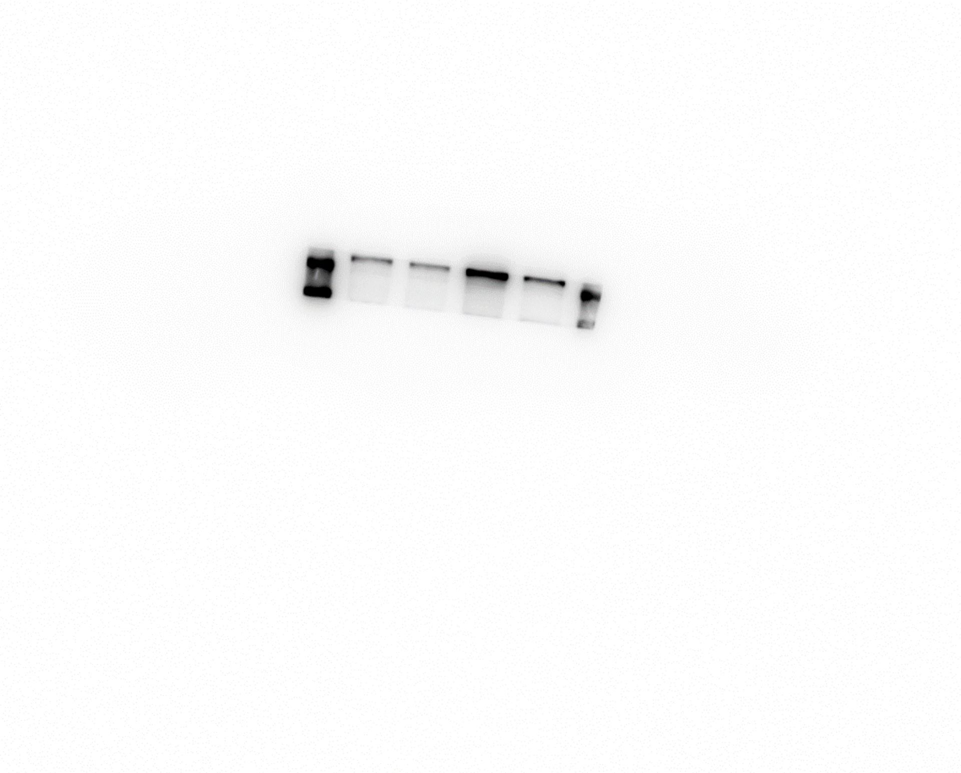

Figure 3C-Collagen-I-2

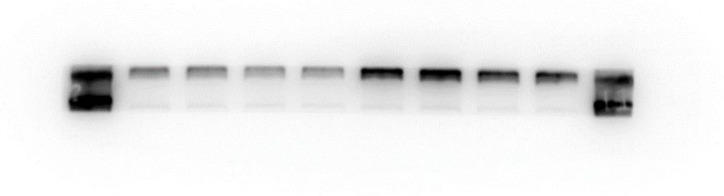

Figure 3C-Fibronectin-1

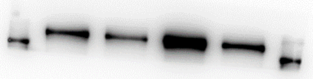

Figure 3C-Fibronectin-2

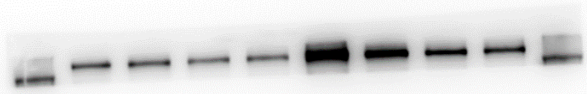

Figure 3C-GAPDH-1

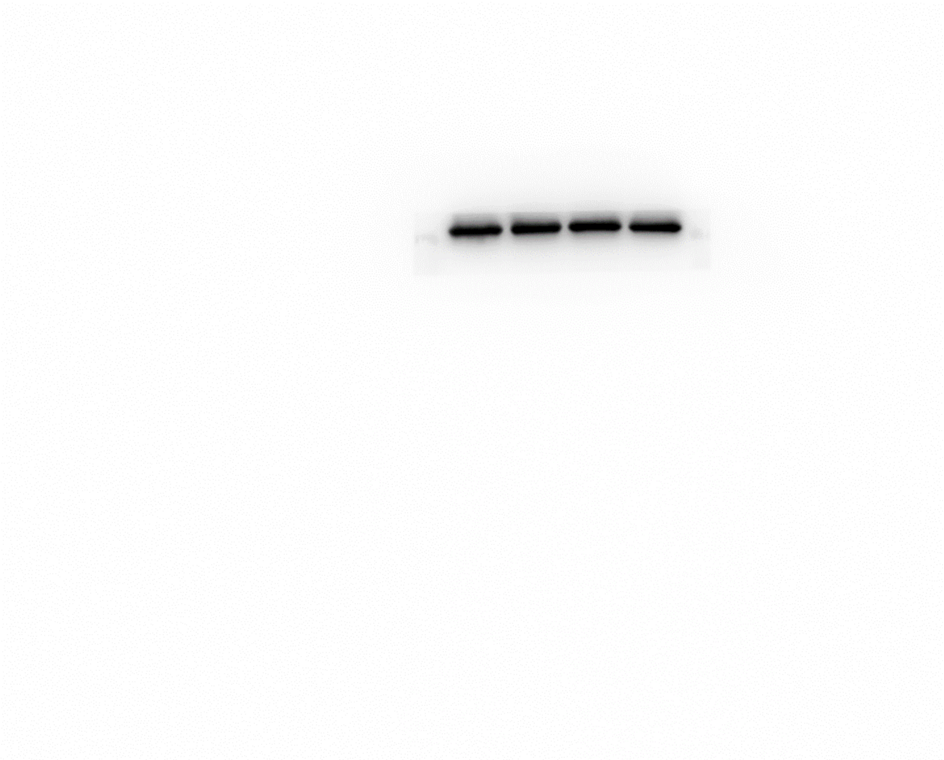

Figure 3C-GAPDH-2

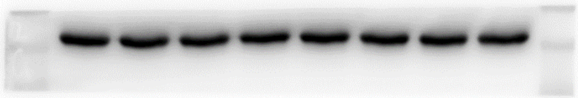

Figure 3C-P-Smad3-1

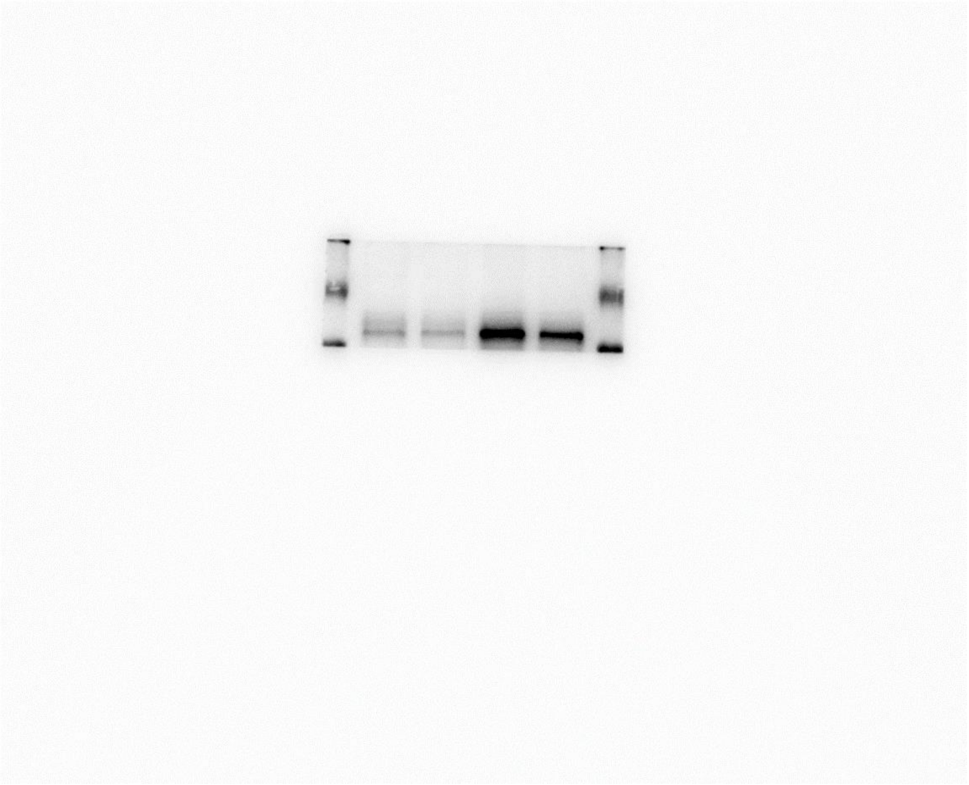

Figure 3C-P-Smad3-2

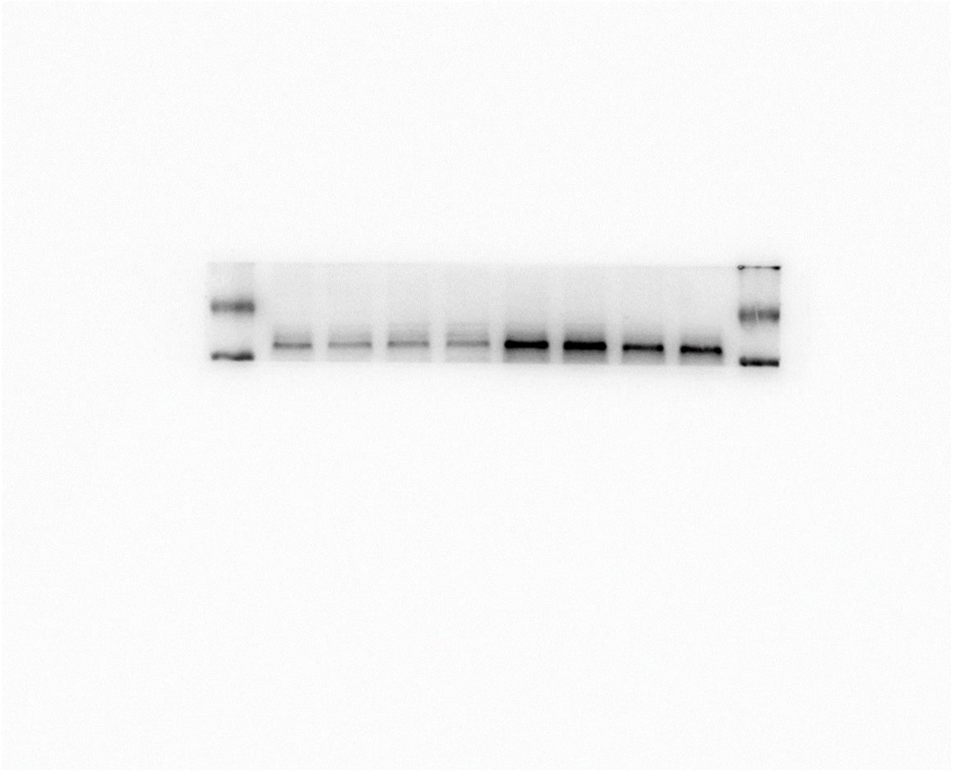

Figure 3C-Smad3-1

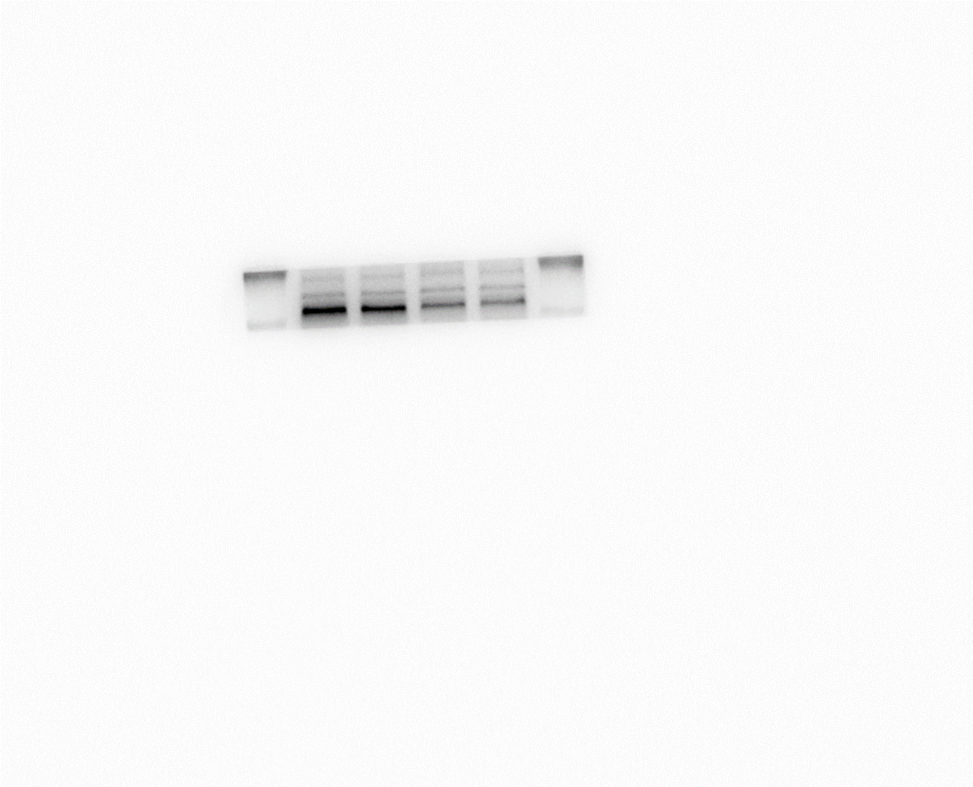

Figure 3C-Smad3-2

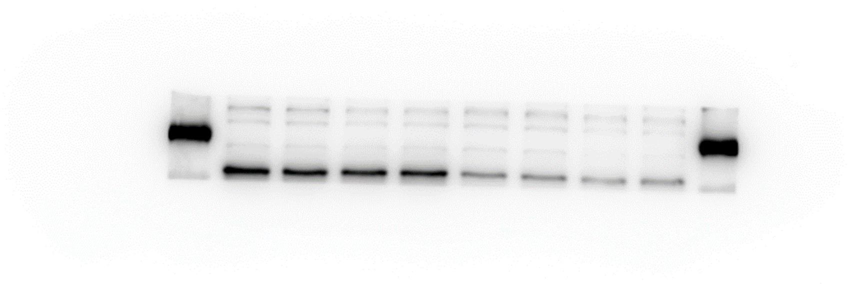

Figure 3C-TGF-β1-1

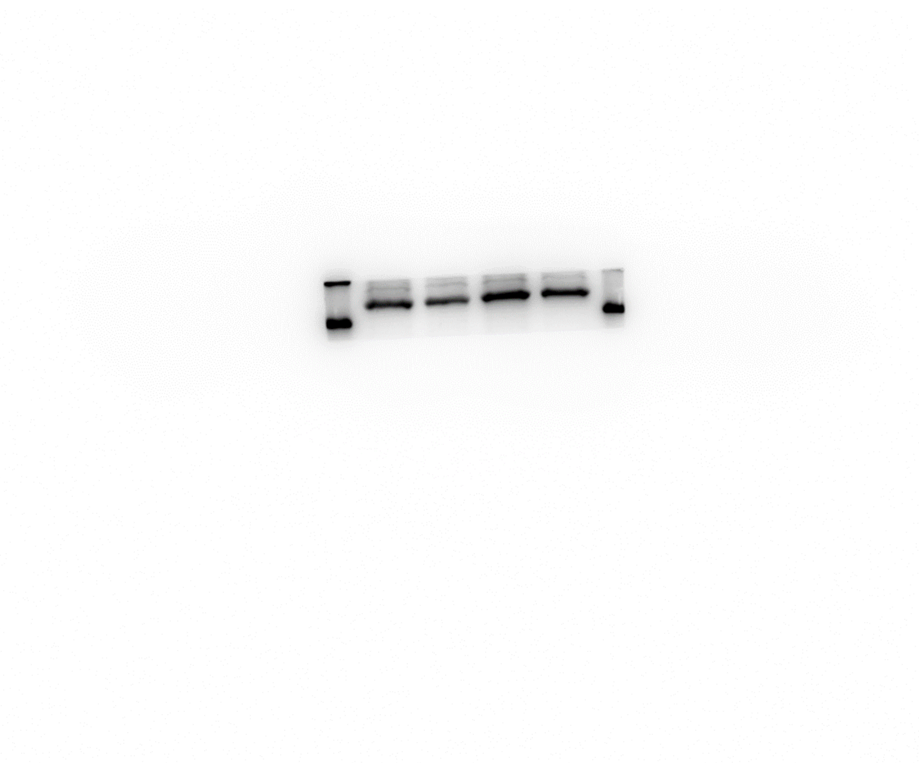

Figure 3C-TGF-β1-2

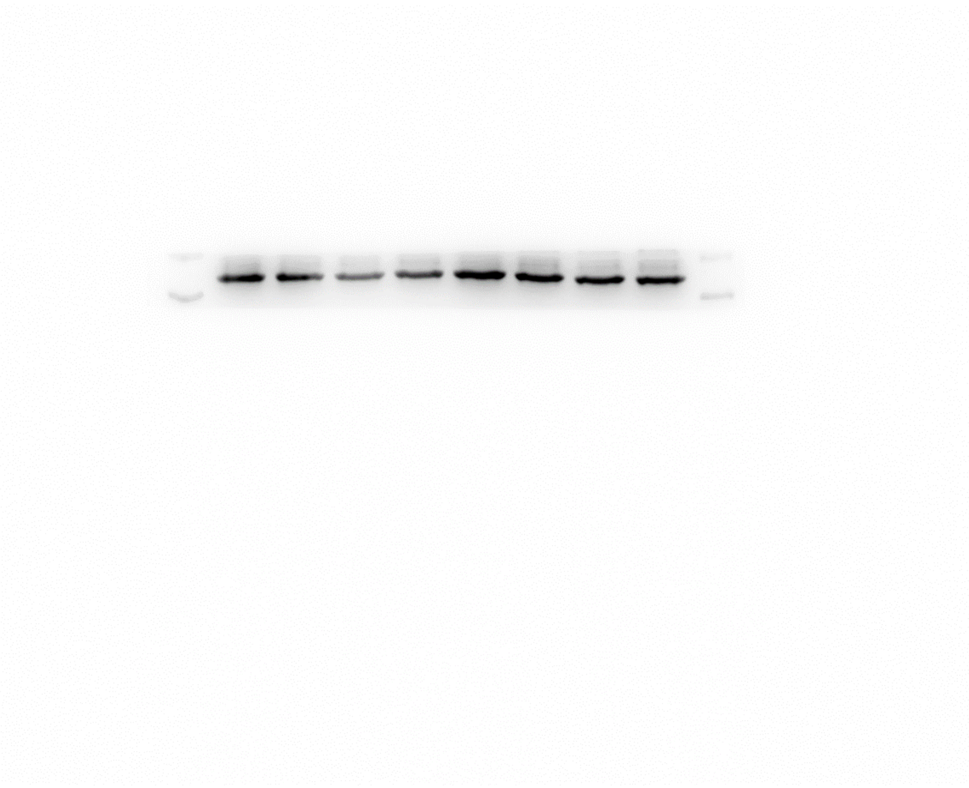

Figure 6B-a-SMA

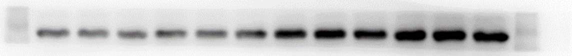

Figure 6B-CDA1

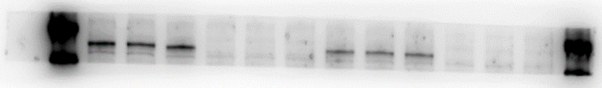

Figure 6B-Collagen I

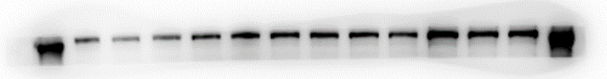

Figure 6B-Fibronectin

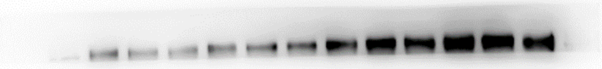

Figure 6B-GAPDH

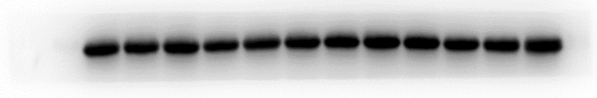

Figure 6B-P-Smad3

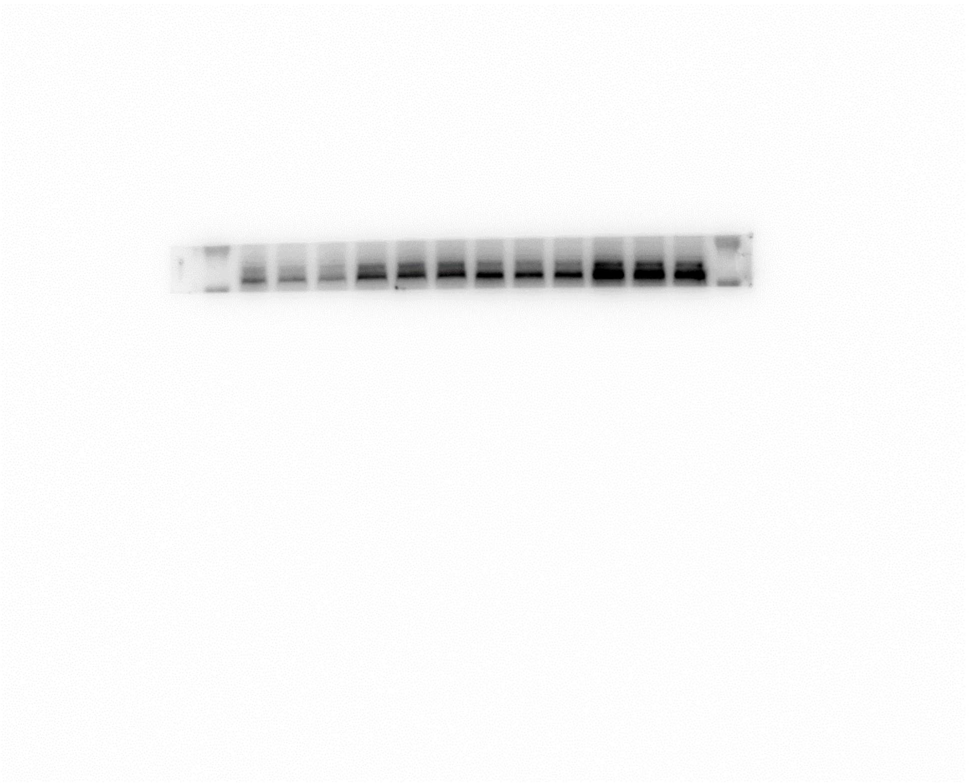

Figure 6B-Smad3

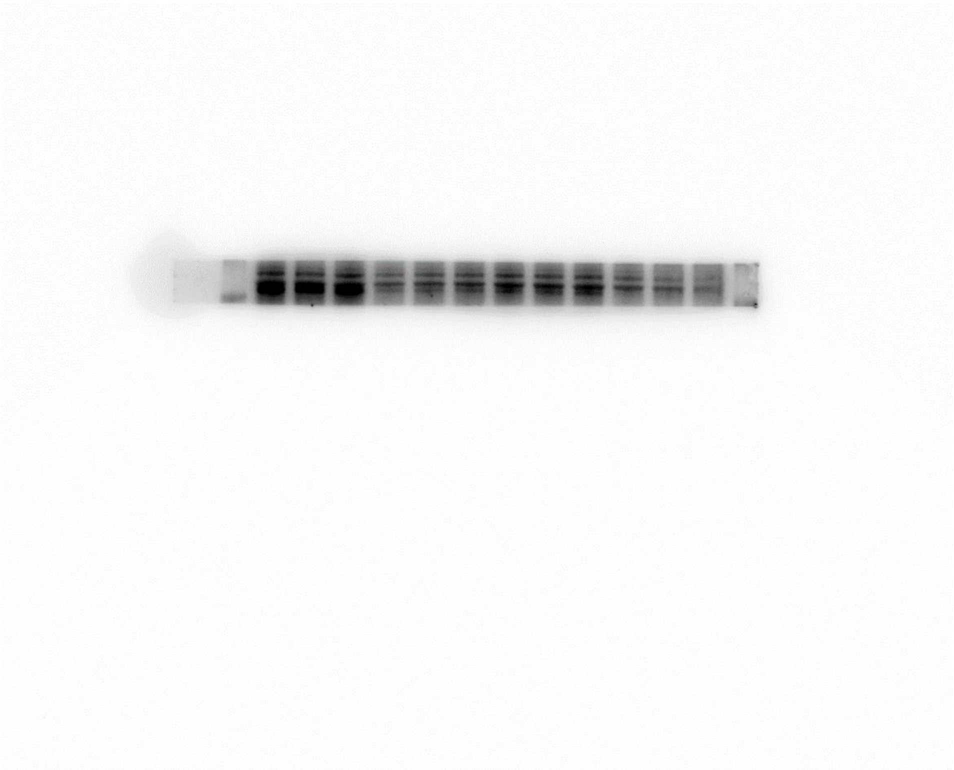

Figure 6B-TGF-β1

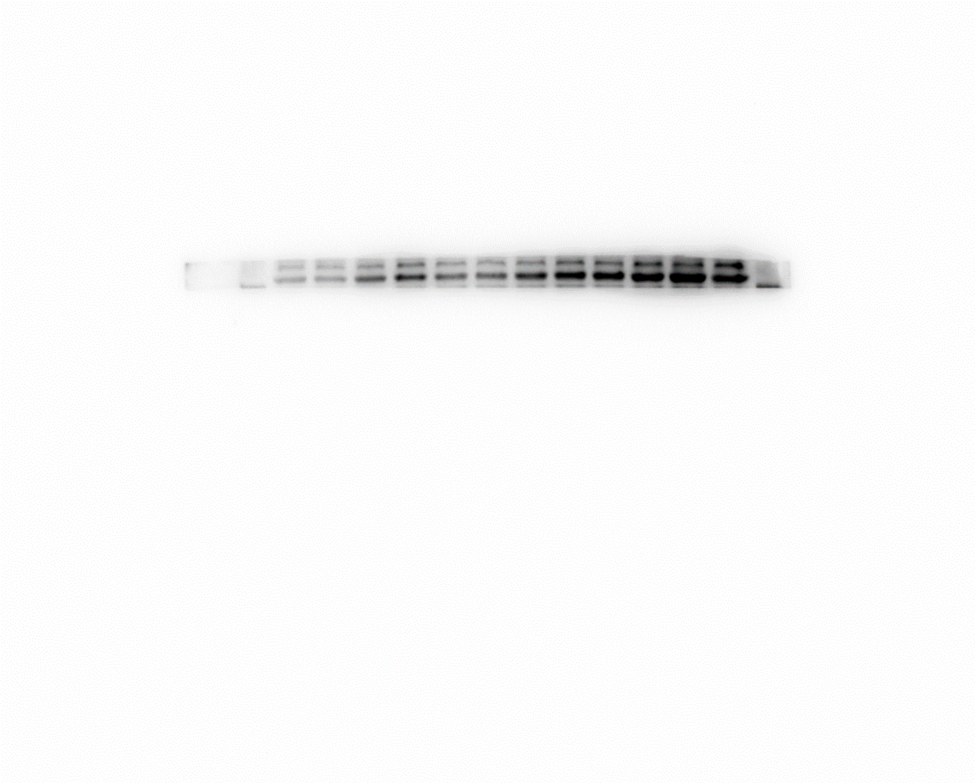

Figure 7D-CDA1-1

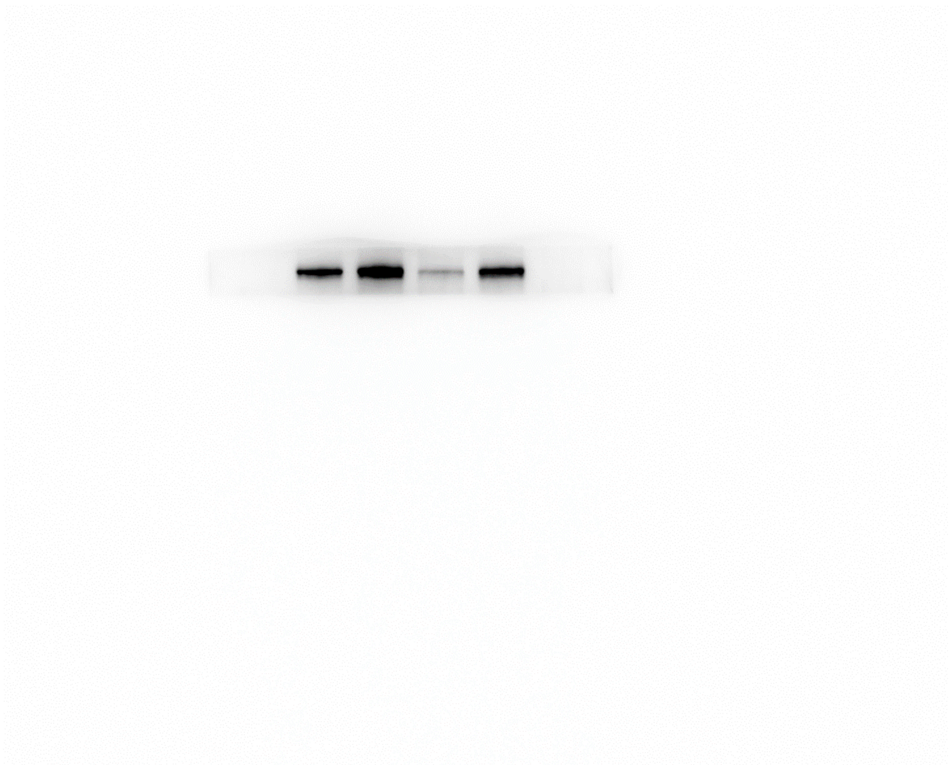

Figure 7D-CDA1-2

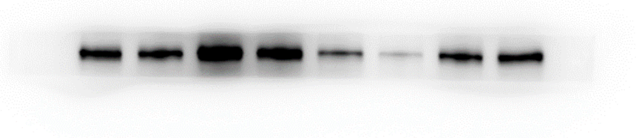

Figure 7D-Collagen I-1

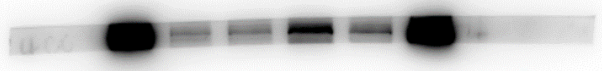

Figure 7D-Collagen I-2

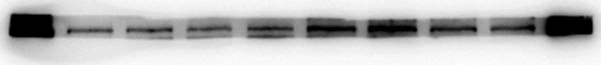

Figure 7D-Fibronectin-1

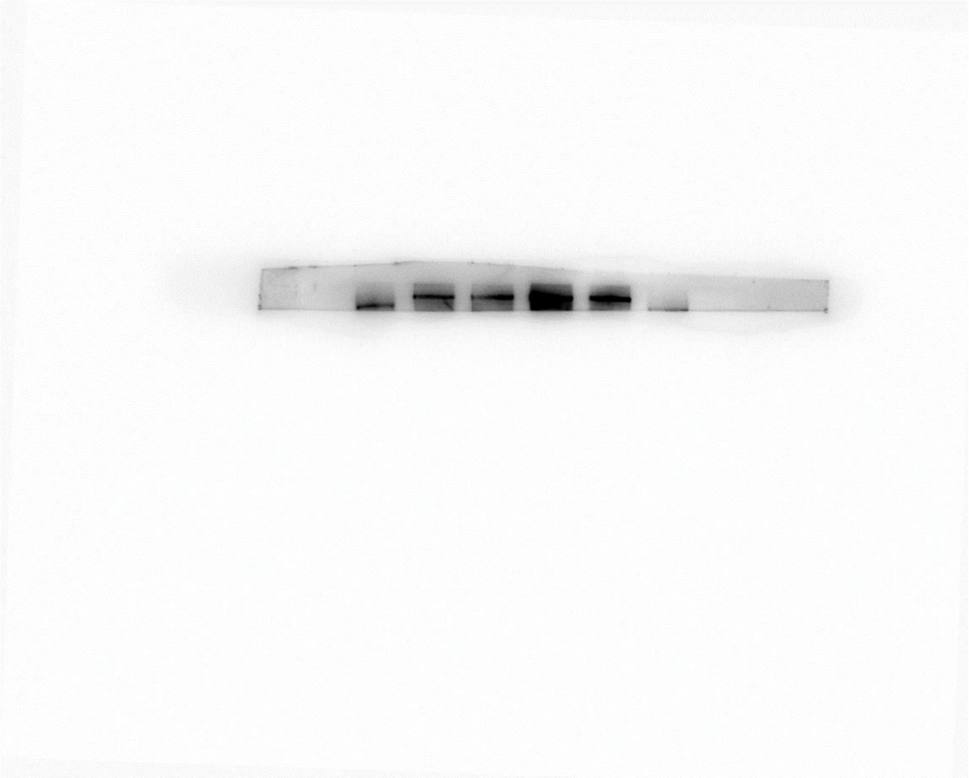

Figure 7D-Fibronectin-2

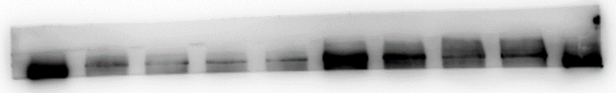

Figure 7D-P-Smad3-1

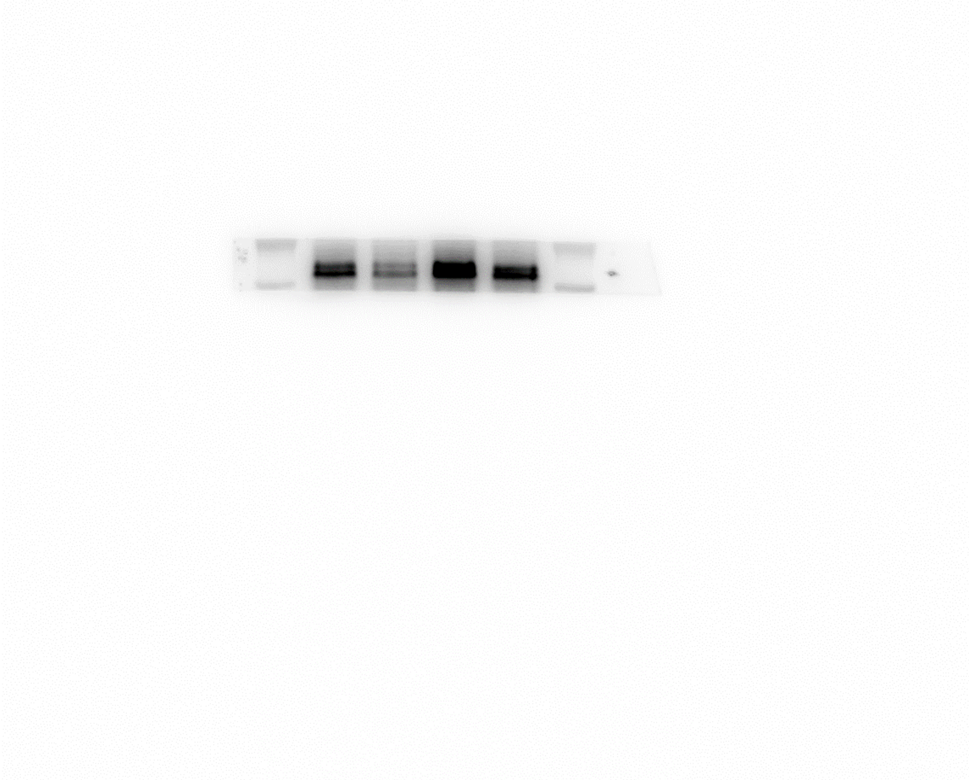

Figure 7D-P-Smad3-2

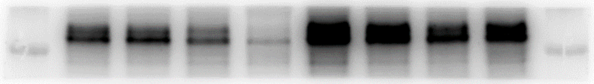

Figure 7D-Smad3-1

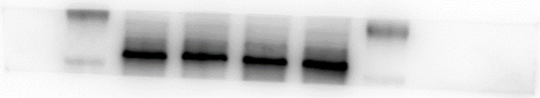

Figure 7D-Smad3-2

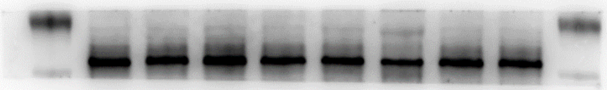

Figure 7D-TGF-β1-1

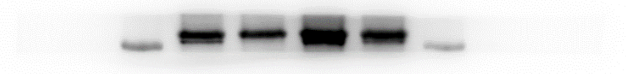

Figure 7D-TGF-β1-2

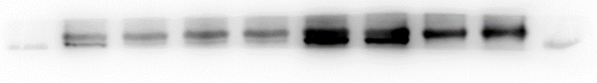

Figure 7D-β-actin-1

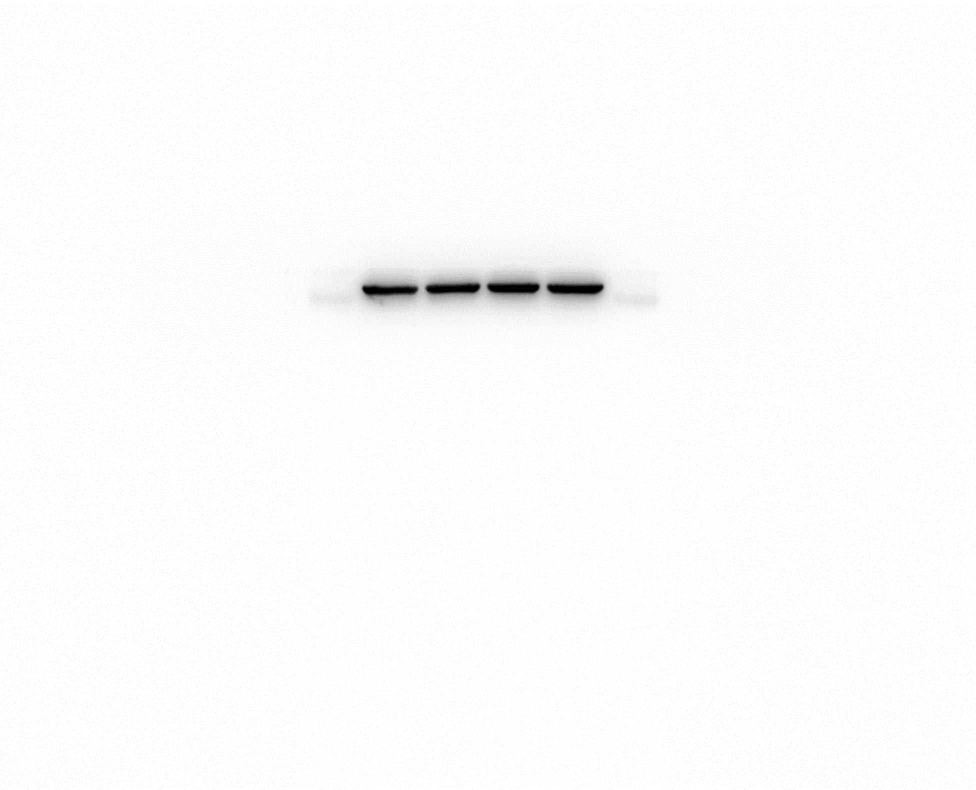

Figure 7D-β-actin-2

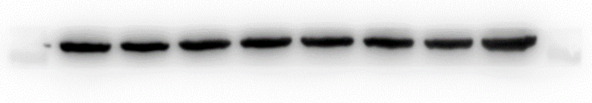

Supplementary Figure 2A-GFP

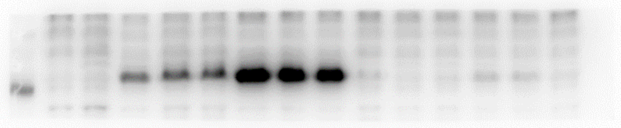

Supplementary Figure 2A-β-actin

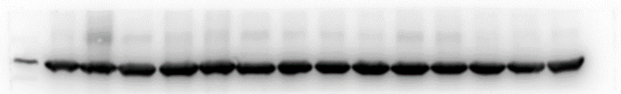

Supplement: Supplementary file 7 — the full length uncropped original western blots [file 41419_2023_5889_MOESM7_ESM.pdf]
